# A clustering of heterozygous missense variants in the crucial chromatin modifier WDR5 defines a new neurodevelopmental disorder

**DOI:** 10.1016/j.xhgg.2022.100157

**Published:** 2022-11-01

**Authors:** Lot Snijders Blok, Jolijn Verseput, Dmitrijs Rots, Hanka Venselaar, A. Micheil Innes, Connie Stumpel, Katrin Õunap, Karit Reinson, Eleanor G. Seaby, Shane McKee, Barbara Burton, Katherine Kim, Johanna M. van Hagen, Quinten Waisfisz, Pascal Joset, Katharina Steindl, Anita Rauch, Dong Li, Elaine H. Zackai, Sarah E. Sheppard, Beth Keena, Hakon Hakonarson, Andreas Roos, Nicolai Kohlschmidt, Anna Cereda, Maria Iascone, Erika Rebessi, Kristin D. Kernohan, Philippe M. Campeau, Francisca Millan, Jesse A. Taylor, Hanns Lochmüller, Martin R. Higgs, Amalia Goula, Birgitta Bernhard, Danita J. Velasco, Andrew A. Schmanski, Zornitza Stark, Lyndon Gallacher, Lynn Pais, Paul C. Marcogliese, Shinya Yamamoto, Nicholas Raun, Taryn E. Jakub, Jamie M. Kramer, Joery den Hoed, Simon E. Fisher, Han G. Brunner, Tjitske Kleefstra

**Affiliations:** 1 Human Genetics Department, Radboud University Medical Center, Nijmegen, the Netherlands; 2 Language & Genetics Department, Max Planck Institute for Psycholinguistics, Nijmegen, the Netherlands; 3 Donders Institute for Brain, Cognition and Behaviour, Radboud University Medical Center, Nijmegen, the Netherlands; 4 Centre for Molecular and Biomolecular Informatics, Radboud Institute for Molecular Life Sciences, Radboud University Medical Center, Nijmegen 6500HB, the Netherlands; 5 The Department of Medical Genetics and Alberta Children’s Hospital Research Institute, Cumming School of Medicine, University of Calgary, Calgary, AB, Canada; 6 Department of Clinical Genetics and School for Oncology and Developmental Biology (GROW-School for Oncology and Reproduction), Maastricht UMC+, Maastricht, the Netherlands; 7 Department of Clinical Genetics, Genetics and Personalized Medicine Clinic, Tartu University Hospital, Tartu, Estonia; 8 Department of Clinical Genetics, Institute of Clinical Medicine, University of Tartu, Tartu, Estonia; 9 Translational Genomics Group, Broad Institute of MIT and Harvard, Cambridge, MA, USA; 10 Genomic Informatics Group, University Hospital Southampton, Southampton, UK; 11 Northern Ireland Regional Genetics Service, Belfast City Hospital, Belfast HSC Trust, Belfast BT9 7AB, UK; 12 Ann and Robert H. Lurie Children’s Hospital and Northwestern University Feinberg School of Medicine, Chicago, IL, USA; 13 Department of Human Genetics, Amsterdam UMC, Vrije Universiteit Amsterdam, Amsterdam, the Netherlands; 14 Medical Genetics, Institute of Medical Genetics and Pathology, University Hospital Basel, Basel, Switzerland; 15 Institute of Medical Genetics, University of Zuirch, Schlieren-Zurich, Switzerland; 16 University Children’s Hospital Zurich, Zurich, Switzerland; 17 Center for Applied Genomics, The Children’s Hospital of Philadelphia, Philadelphia, PA, USA; 18 Division of Human Genetics, Department of Pediatrics, The Children’s Hospital of Philadelphia, Philadelphia, PA, USA; 19 Department of Pediatrics, Perelman School of Medicine at the University of Pennsylvania, Philadelphia, PA, USA; 20 Department of Neuropediatrics, Developmental Neurology and Social Pediatrics, Centre for Neuromuscular Disorders in Children, University Hospital Essen, University of Duisburg-Essen, Essen, Germany; 21 Children’s Hospital of Eastern Ontario Research Institute, Ottawa, ON, Canada; 22 Department of Neurology, University Hospital Bergmannsheil, Heimer Institute for Muscle Research, 44789 Bochum, Germany; 23 Institute of Clinical Genetics and Tumor Genetics, Bonn, Germany; 24 Department of Pediatrics, ASST Papa Giovanni XXIII, Bergamo, Italy; 25 Laboratory of Medical Genetics, ASST Papa Giovanni XXIII, Bergamo, Italy; 26 Pediatric Neurological Unit and Epilespy Center, Fatebenefratelli Hospital, Milan, Italy; 27 Newborn Screening Ontario, Children’s Hospital of Eastern Ontario and Children’s Hospital of Eastern Ontario Research Institute, Ottawa, ON, Canada; 28 CHU Sainte-Justine Research Center, Montreal, QC H3T 1C5, Canada; 29 Sainte-Justine Hospital, University of Montreal, Montreal, QC H3T 1C5, Canada; 30 GeneDx, Gaithersburg, MD 20877, USA; 31 Division of Plastic Surgery, Department of Surgery, The Children’s Hospital of Philadelpia, Philadelphia, PA, USA; 32 Children’s Hospital of Eastern Ontario Research Institute, Division of Neurology, Department of Medicine, the Ottawa Hospital, Brain and Mind Research Institute, University of Ottawa, Ottawa, Canada; 33 Lysine Methylation and DNA Damage Laboratory, Institute of Cancer and Genomic Sciences, University of Birmingham, Birmingham, UK; 34 North-West Thames Regional Genetics Service, North West London Hospitals NHS Trust, Munroe-Meyer, Harrow, UK; 35 Institute for Genetics and Rehabilitation, University of Nebraska Medical Center, Omaha, NE, USA; 36 Victorian Clinical Genetics Services, Murdoch Children’s Research Institute, Melbourne, VIC, Australia; 37 Department of Paediatrics, University of Melbourne, Melbourne, VIC, Australia; 38 Broad Center for Mendelian Genomics, Program in Medical and Population Genetics, Broad Institute of MIT and Harvard, Cambridge, MA, USA; 39 Department of Molecular and Human Genetics, Baylor College of Medicine, Houston, TX, USA; 40 Jan and Dan Duncan Neurological Research Institute, Texas Children’s Hospital, Houston, TX 77030, USA; 41 Department of Biochemistry and Molecular Biology, Faculty of Medicine, Dalhousie University, Halifax, NS, Canada; 42 International Max Planck Research School for Language Sciences, Max Planck Institute for Psycholinguistics, Nijmegen, the Netherlands; 43 Donders Institute for Brain, Cognition & Behaviour, Radboud University, Nijmegen, the Netherlands

**Keywords:** WDR5, COMPASS, neurodevelopmental disorders, intellectual disability, Mendelian disorders, multiple congenital abnormalities, missense variants, next generation sequencing, *de novo* variants

## Abstract

WDR5 is a broadly studied, highly conserved key protein involved in a wide array of biological functions. Among these functions, WDR5 is a part of several protein complexes that affect gene regulation via post-translational modification of histones. We collected data from 11 unrelated individuals with six different rare *de novo* germline missense variants in *WDR5*; one identical variant was found in five individuals and another variant in two individuals. All individuals had neurodevelopmental disorders including speech/language delays (n = 11), intellectual disability (n = 9), epilepsy (n = 7), and autism spectrum disorder (n = 4). Additional phenotypic features included abnormal growth parameters (n = 7), heart anomalies (n = 2), and hearing loss (n = 2). Three-dimensional protein structures indicate that all the residues affected by these variants are located at the surface of one side of the WDR5 protein. It is predicted that five out of the six amino acid substitutions disrupt interactions of WDR5 with RbBP5 and/or KMT2A/C, as part of the COMPASS (complex proteins associated with Set1) family complexes. Our experimental approaches in *Drosophilamelanogaster* and human cell lines show normal protein expression, localization, and protein-protein interactions for all tested variants. These results, together with the clustering of variants in a specific region of WDR5 and the absence of truncating variants so far, suggest that dominant-negative or gain-of-function mechanisms might be at play. All in all, we define a neurodevelopmental disorder associated with missense variants in *WDR5* and a broad range of features. This finding highlights the important role of genes encoding COMPASS family proteins in neurodevelopmental disorders.

## Teaser

By identifying and characterizing 11 individuals with rare *de novo* variants in *WDR5,* we defined a new Mendelian disorder associated with missense variants in this gene. The affected individuals all had neurodevelopmental disorders with a broad spectrum of additional features, including epilepsy, aberrant growth parameters, and skeletal and cardiac abnormalities.

## Main text

WDR5 is a well-studied, highly conserved, and ubiquitously expressed protein^1^^,^^2^^,^^3^ with impacts on many crucial developmental pathways as part of several different multiprotein complexes.^3^^,^^4^ The indispensable function of WDR5 is illustrated by its high evolutionary conservation. Even simple multicellular organisms such as *Trichoplax adhaerens* have a protein with around 90% similarity to the 334 amino acids of the human ortholog.^1^^,^^3^ Most of the protein complexes that WDR5 participates in affect gene regulation via post-translational modification of histones, e.g., the complex proteins associated with Set1 (COMPASS) family complexes,^5^^,^^6^ the non-specific lethal (NSL) complex,^7^ the Ada2a-containing (ATAC) complex,^8^ and the nucleosome remodeling and deacetylase (NuRD) complex.^9^ In addition to influencing cellular processes via protein-protein interactions, WDR5 is able to bind to >1,000 different endogenous RNA molecules.^10^ WDR5 has an important role in embryonic stem cell (ESC) self-renewal and maintenance of a pluripotent state.^11^^,^^12^ More recent studies have linked WDR5 to a newly discovered genetic compensation mechanism called nonsense-induced transcriptional compensation.^13^ Moreover, WDR5 has been identified as a critical co-factor for retinoic acid signaling,^14^ and directly interacts with p53 to regulate mouse ESC stem cell fate in a p53-dependent manner.^15^ While the biological functions of the WDR5 protein have been studied from numerous angles, little is known about the impact of germline *WDR5* variants in humans*.*

**Figure 1:**
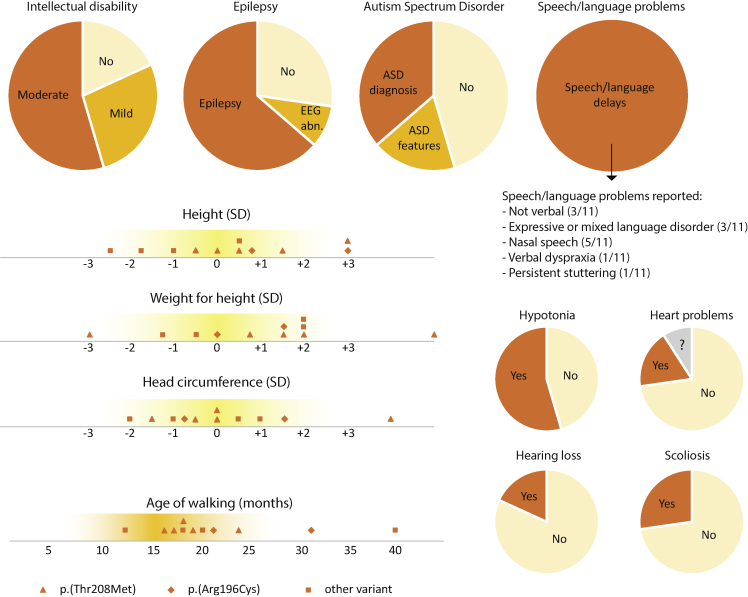
Clinical features reported in individuals with *WDR5* variants Graphical overview of clinical features reported in 11 individuals with *WDR5* missense variants. Growth parameters are shown as standard deviations to the mean for a certain age. All graphs include data for 11 individuals (N = 11). EEG abn., EEG abnormalities. A more detailed overview of clinical features can be found in Table S1.

The initial finding of a *de novo* missense variant (c.623C>T, p.(Thr208Met)) in *WDR5* in a proband with childhood apraxia of speech^16^ prompted us to investigate the effects and possible pathogenicity of rare germline variants in this gene. Using the GeneMatcher database^17^ and other international collaborations, we collected clinical information on 11 unrelated individuals with rare *de novo* germline variants in *WDR5* that was collated from several clinical exome or genome sequencing studies (supplemental materials and methods). In these 11 individuals, six different missense variants were reported in *WDR5*: c.505C>G (p.(Ala169Pro)), c.586C>T (p.(Arg196Cys)), c.602C>T (p.(Ala201Val)), c.623C>T (p.(Thr208Met)), c.637G>A (p.(Asp213Asn)), and c.734A>G (p.(Lys245Arg)) (Table S1). All individuals had neurodevelopmental disorders with a spectrum of overlapping additional features (Figure 1; Table S1). Intellectual disability (ID) was present in 9/11 individuals, with a severity ranging from moderate ID (IQ 35–50, six individuals) to mild ID (IQ 50–70, three individuals). Speech delays were reported in all individuals, including nasal speech, developmental language disorders, verbal dyspraxia, and persistent stuttering. Three individuals remained nonverbal. All but one individual had delays in motor development, and hypotonia was reported in six individuals. Two individuals had ataxia. Seven individuals were diagnosed with different forms of epilepsy. Concerning the behavioral phenotype, four individuals had an autism spectrum disorder (ASD) diagnosis, and two individuals were diagnosed with attention deficit hyperactivity disorder (ADHD).

**Figure 2:**
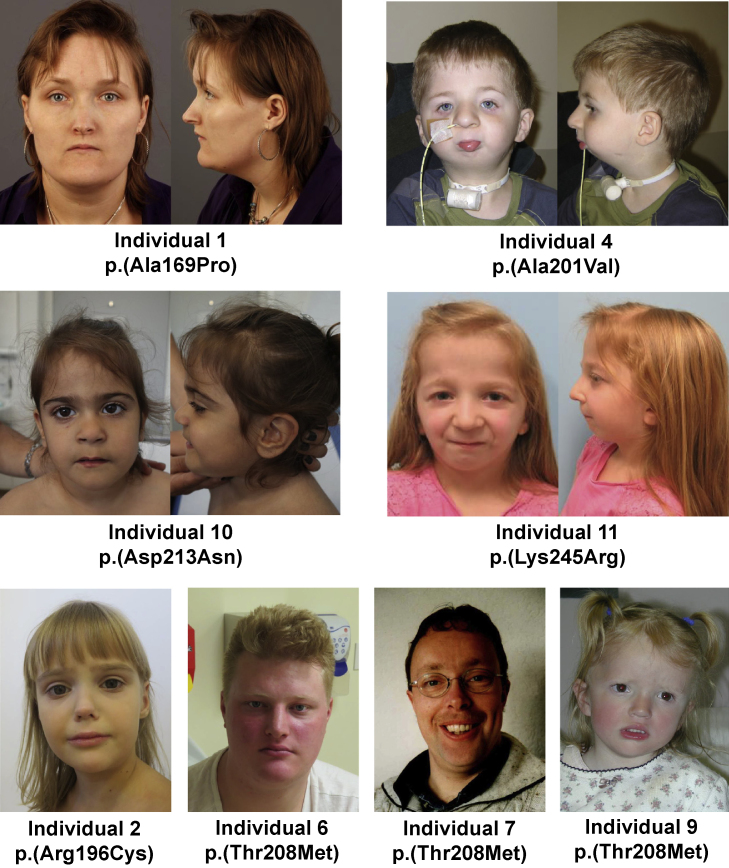
Facial features in individuals with six different *WDR5* variants Facial images of eight individuals with a heterozygous *WDR5* variant. Several overlapping facial features are seen, such as a bulbous nasal tip (individuals 2, 4, and 10), low-set, posteriorly rotated, and/or dysplastic ears (individuals 2, 4, 7, and 10), ptosis (individual 11), and thin upper lip vermilion (individuals 4, 10, and 11). In addition, individuals 4 and 11 have severe micrognathia, a small mouth, and down-slanting palpebral fissures.

The individuals with *WDR5* variants showed divergent growth parameters (Figure 1). No clear correlation between height, weight, and head circumference was observed (Table S1), with the exception of two individuals (individuals 2 and 6) with a generalized overgrowth phenotype. Different abnormalities of the skeleton and limbs were present in a subset of individuals: scoliosis, kyphosis (with hemivertebra L5), bilateral clubfeet, and hemihypertrophy of one leg. In two individuals, heart abnormalities were reported: cardiac arrhythmias and decompensated heart failure requiring surgery in one individual, and left ventricular noncompaction cardiomyopathy in another individual. Four individuals were reported with frequent infections. However, one of these individuals had a combined immunodeficiency likely caused by a pathogenic missense variant in TNFRSF13B (Table S1). Overlapping facial features included a bulbous nasal tip, low-set, posteriorly rotated, and/or dysplastic ears, ptosis, and thin lip vermilion (Figure 2). Two individuals (4 and 11) had distinct facial features, with severe micrognathia (requiring tracheostomy in one), a small mouth, and prominent down-slanting palpebral fissures. Both had conductive hearing loss, too, a feature not reported in any of the other individuals. The variable expressivity of associated features and severity of symptoms is prominent, and we did not observe any clear genotype-phenotype correlation between specific variants and specific phenotypes. Even in five individuals with the exact same missense variant, p.(Thr208Met), a different clinical presentation was seen, e.g., borderline versus moderate ID and normal growth parameters versus a generalized overgrowth phenotype. Clinical features reported in individuals in our cohort are described in more detail in Table S1.

**Figure 3:**
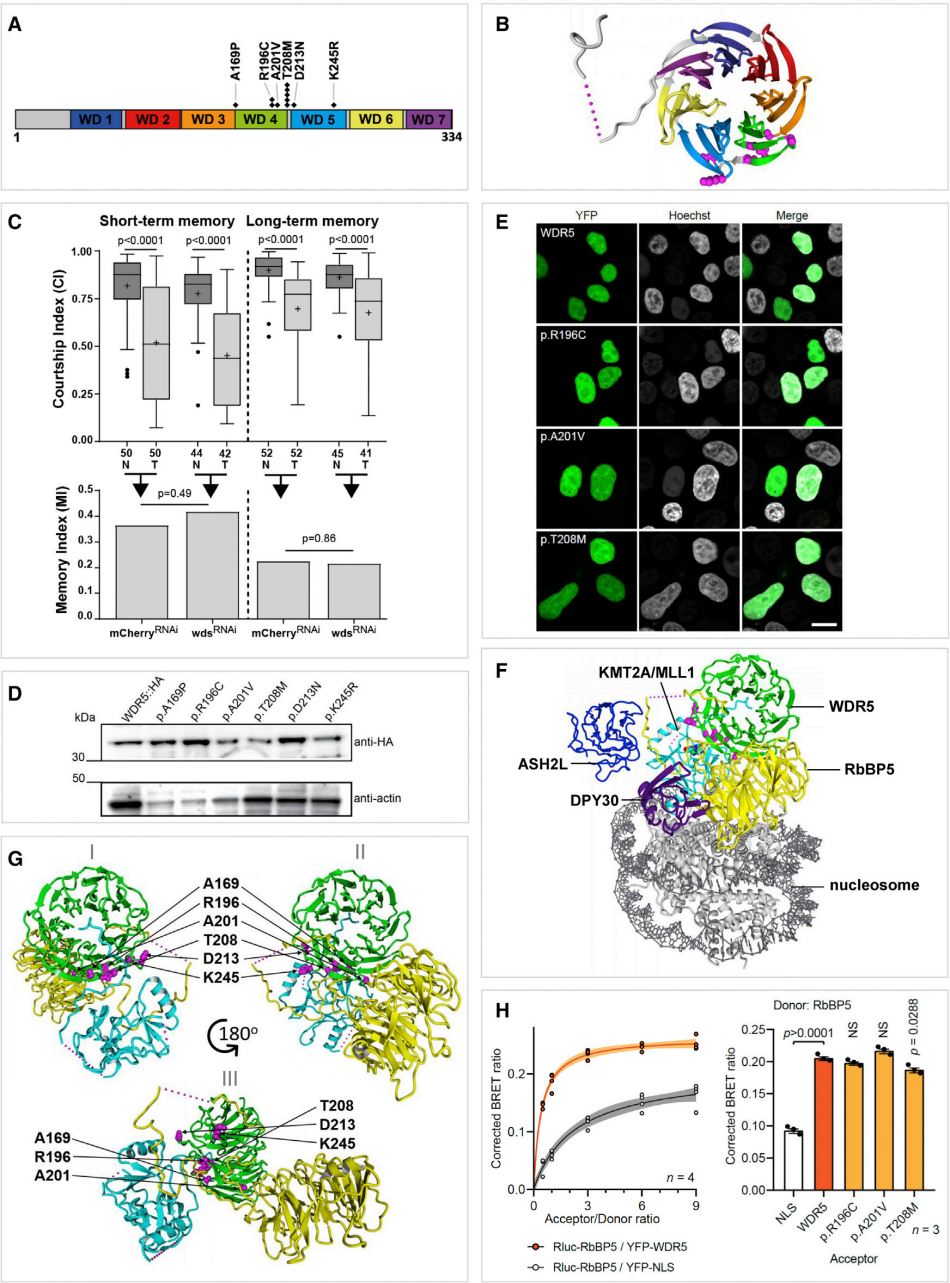
*In silico, in vitro*, and *in vivo* studies of the effect of *WDR5* missense variants (A) Linear structure of WDR5 protein (334 amino acids) with the seven different WD40 domains and all identified missense variants shown; in total, six different missense variants were found in 11 individuals: one variant (p.(Thr208Met)) was found in five unrelated individuals and another variant (p.(Arg196Cys)) in two individuals. (B) Three-dimensional visualization of WDR5 (PDB: 2GNQ); locations of the amino acids involved in missense variants are shown with magenta balls. Colors of the different domains match with the colors used in (A). (C) Courtship memory was assessed in mushroom-body-specific wds RNAi knockdown flies (wds^RNAi^) compared with controls expressing an RNAi against mCherry (mCherry^RNAi^). Boxplots show the distribution of courtship indices (CIs) for naive (N) and trained (T) flies (top panel). Memory was observed when a significant reduction in CI occurred between naive and trained conditions (Mann-Whitney test). + indicates the mean. N is indicated along the x axis. Bar graphs show the memory indices (MIs), which are single values derived from the above CIs (indicated by arrows) according to the formula: MI = ($\bar{\text{X}}$ CI_naive_ − $\bar{\text{X}}$ CI_trained_)/ $\bar{\text{X}}$ CI_naive_. MIs were consistent between controls and wds^RNAi^ lines (randomization test, 10,000 bootstrap replicates). (D) Detection of WDR5 reference and variant proteins in adult flies by western blot. Representative bands for HA-tagged WDR5 reference and variant proteins at 36.6 kDa (top) along with the actin loading control 41 kDa (bottom). *UAS-WDR5::HA* reference and variant transgenes were expressed ubiquitously using *Actin-Gal4*. (E) Direct fluorescence micrographs of nuclei of HEK293T/17 cells expressing YFP-WDR5 fusion proteins (green). Nuclei were stained with Hoechst 33342 (blue). Scale bar: 10 μm. (F) WDR5 (green) is shown as part of the core COMPASS complex, with RbBP5 (yellow), ASH2L (blue), DPY30 (purple), and KMT2A (cyan) (PDB: 6KIV). The nucleosome is shown in gray. The locations of affected amino acids in individuals with missense variants are shown with magenta balls. (G) WDR5 (green; p.33–332) is shown together with RbBP5 (yellow; p.1–380) and KMT2A (cyan; p.3764–3969) as part of the core COMPASS complex (PDB: 6KIV). The locations of affected amino acids in individuals with missense variants are shown with magenta balls from three different angles facing the WIN site (I), the WBM site (II), and a side between WIN and WBM (III). (H) BRET assays for WDR5-RbBP5 interaction in live cells. Left, mean BRET saturation curves ±95% confidence interval fitted using a nonlinear regression equation assuming a single binding site (n = 4; y = BRETmax ∗ x/(BRET50/x); GraphPad) showing a strong BRET signal for Rluc-RbBP5 with YFP-WDR5. Right, corrected BRET values measured with an acceptor/donor ratio of 1:1 (n = 3, one-way ANOVA and post-hoc Bonferroni test). NLS, YFP fused to a C-terminal nuclear localization signal as control protein. p values show significance for the comparison of WDR5 variant with the WDR5 wild-type.

Regarding the molecular aspects of *WDR5*, we found six different missense variants in 11 unrelated individuals: the p.(Thr208Met) variant was reported in five individuals and the p.(Arg196Cys) in two individuals. All variants were confirmed to be *de novo*, and none were in the gnomAD database,^18^ showing that these variants are extremely rare on a population level. We used *in silico* prediction programs to evaluate pathogenicity for the variants, and while all CADD scores were above 22, SIFT and PolyPhen-2 predicted only three to be pathogenic: p.(Ala169Pro), p.(Arg196Cys), and p.(Thr208Met) (Table S1). Using a linear model of WDR5, we found all missense variants to be located within or flanking the fourth and the fifth WD40 domain of WDR5 (Figure 3A). As a member of the WD40 repeat protein family, WDR5 has seven WD40 domains that each form a propeller-like wing^19^ of the final “barrel” -shaped protein (Figure 3B).

We used different experimental approaches in fruit flies (*Drosophila melanogaster*) and human cell lines to assess possible pathogenic effects of *WDR5* variants. Previous studies showed that the histone methyltransferase subunits of the COMPASS complexes are required in *Drosophila* memory neurons of the mushroom body for normal memory function.^20^^,^^21^ Here, we used the same approach to test mushroom-body specific knockdown of the fly WDR5-ortholog *will die slowly* (*wds*) (supplemental materials and methods). While *wds* was efficiently knocked down in the transgenic RNAi line (wds^RNAi^) that we used, we did not observe any differences in short- or long-term memory outcomes upon *wds* knockdown compared with controls (mCherry^RNAi^) (Figure 3C). This suggests that memory neurons are more resilient to loss of *wds/WDR5* than to loss of COMPASS complex enzymatic subunits. In addition, we assessed the expression of reference or variant human WDR5 proteins tagged with C′-3xHA tag (WDR:HA) driven with a ubiquitous driver in transgenic *Drosophila* strains using western blot (supplemental materials and methods). No difference was observed for the protein expression levels of any of the six examined WDR5 proteins with amino acid substitutions (p.(Ala169Pro), p.(Arg196Cys), p.(Ala201Val), p.(Thr208Met), p.(Asp213Asn), and p.(Lys245Arg)) compared with wild-type WDR5 (Figure 3D). Consistently, three WDR5 variant proteins (p.(Arg196Cys), p.(Ala201Val), and p.(Thr208Met)) did not show any differences in subcellular localization (Figure 3E) or protein expression levels (Figure S1) compared with wild-type WDR5 when overexpressed as yellow fluorescent protein (YFP)-fusion proteins in HEK293/T17 cells (supplemental materials and methods). Taken together, both *in vivo* and *in vitro* studies showed that the missense variants functionally assessed in our study lead to stable and normally expressed WDR5 proteins. This, combined with the absence of truncating variants in our cohort, argues against a sole loss-of-function effect as the underlying pathogenic mechanism. The fact that all variants found in affected individuals are missense variants and the clustering and recurrence of these variants at specific positions suggest that other mechanisms might be at play, such as dominant-negative or gain-of-function effects.

Through three-dimensional protein structure analysis, we determined that the amino acid residues affected by the six missense variants cluster together in the three-dimensional protein structure of WDR5, and more specifically on the surface of one side of the encoded protein. An interesting hypothesis for pathogenicity of the missense variants is that the amino acid substitutions disrupt specific interactions with other proteins. A landscape of intolerance for genetic variation in the *WDR5* gene visualized in the three-dimensional structure of the encoded protein shows that while WDR5 is generally intolerant to missense variants, residues that interact with other proteins have highest intolerance for normal variation (Figure S2). WDR5 is able to act as a molecular adapter to facilitate protein-protein interactions^3^ using two distinct binding sites identified in previously performed co-precipitation experiments: the “WDR5-interacting” (WIN) site^22^^,^^23^^,^^24^ and the “WDR5-binding motif” (WBM) site^24^^,^^25^ located on opposite sides of the protein. The missense variants in our study were not located in the vicinity of these two most well-studied binding locations. However, recently published cryo-electron microscopy three-dimensional structures of the COMPASS complexes revealed a region, located between the WIN and WBM binding sites, that is involved in the interaction with RbBP5 and histone-lysine methylase (KMT) enzymes in these complexes.^26^ Five out of the six missense variants in our cohort map within this RbBP5/KMT interaction region. Based on the three-dimensional structure analysis of the COMPASS complexes, p.(Ala169Pro) and p.(Asp213Asn) are predicted to affect the WDR5 interaction with KMT enzymes, and p.(Ala201Val) and p.(Thr208Met) are predicted to affect the interaction with the RbBP5 enzymes, while p.(Arg196Cys) most likely influences the interaction with both enzymes (Figure 3G). The effects of the p.(Lys245Arg) variant cannot be predicted using the currently available three-dimensional structures. A detailed description of the predicted effects of all variants, from the perspective of the structural modeling analyses, is provided in Note S2.

WDR5 is a crucial core protein within the COMPASS complex family: it is essential for complex assembly and activity.^27^^,^^28^ In this context, it is important to note that the detailed three-dimensional structures used for these analyses are unfortunately only available for the COMPASS complex and not for all other complexes and interactions in which WDR5 is involved. Therefore, it remains unclear whether the predicted disruptive effects on WDR5 interactions are specific to those with RbBP5/KMT2 or if interactions with other molecules might also be disturbed. Based on three-dimensional protein structure analysis of COMPASS complexes, it seems that differently composed COMPASS complexes make use of different interaction surfaces of WDR5. Some variants might therefore disrupt interactions in only one specific complex. As WDR5 seems to act as an “adapter” protein, forming links between different molecules, disruption of protein-protein interactions within the complex might have important effects on complex activity.

As a functional follow up, we used bioluminescence resonance energy transfer (BRET) assays in live cells and were able to confirm the WDR5-RbBP5 interaction (Figure 3H). However, we could not demonstrate a biologically significant disruption of the interaction between WDR5 missense variants (p.(Arg196Cys), p.(Ala201Val), and p.(Thr208Met)) and RbBP5 (Figure 3H). While this result does not support the findings of our protein structure analyses, which predict a disruption of the interaction with one of the COMPASS family protein complex members, it does not exclude possible effects of missense variants on the formation and composition of the COMPASS complex or an altered activity of the complex due to the missense variants. In our BRET assays, we investigated a single protein-protein interaction of heterologously expressed fluorescently tagged proteins, and we may not have been able to detect subtle shifts in WDR5-RbBP5 interaction dynamics or possible changes in the composition of the protein complexes involved.

Using three-dimensional structure analyses, we were not able to predict a likely pathogenic mechanism for the p.(Lys245Arg) variant. One hypothesis to explain pathogenicity of this variant could be that the variant affects a so-far-uncharacterized interaction site with RbBP5 or KMT2A/C, as a comparison of available three-dimensional structures between human KMT2A and yeast COMPASS complex suggests even more extensive interaction surfaces between WDR5 and histone methylases (Figure S3). Another hypothesis is that the p.(Lys245Arg) variant affects the interaction with other molecules that are not involved in the COMPASS complex.

In addition to the 11 individuals with missense variants in our study, we identified a *de novo* intronic variant in *WDR5* affecting a canonical splice site (c.742-2del) in an individual with multiple skeletal abnormalities, a cleft palate, acquired microcephaly, short stature (−2 SD), and normal development at 4 years of age (Table S1; Note S1). The skeletal abnormalities included right radial hypoplasia, absent right thumb, four metacarpals of the left hand, hypoplastic thumb bones, soft tissue syndactyly 1-2 of the left hand, and T7 butterfly vertebra with normal lower extremities. Of note, this patient also had a left ventricular noncompaction cardiomyopathy, a rare cardiac abnormality also present in individual 10, with missense variant p.(Asp231Asn). While five different *in silico* splice prediction tools all predicted a loss of the acceptor site of the 12^th^ exon of WDR5, the consequences at mRNA and protein levels are unclear. Three of the splice prediction tools predicted the creation of a new acceptor site 9 bp upstream from the current acceptor site, resulting in an in-frame loss of three amino acids (p.248–250) (Note S1). Interestingly, these three amino acids are located on the surface of WDR5 in the region that interacts with RbBP5. All in all, it is likely that this *de novo* variant affecting a canonical splice site is a pathogenic variant, but it remains unclear how the effect of this splice variant relates to the effect of the missense variants reported in our study.

Our study represents the characterization of multiple probands with a Mendelian disorder associated with germline variants in *WDR5*. It is worth mentioning that, beyond the cases described here, one additional *de novo* variant in *WDR5* has been reported in the literature: a p.(Lys7Gln) variant, found in a child with a conotruncal heart defect with a right aortic arch.^29^ This missense variant is located in the N-terminal tail of WDR5, an intrinsically disordered region of the protein (not available for three-dimensional protein structure analysis), which is not involved in the beta-propeller structure of WDR5, and has been shown to be dispensable for COMPASS complex assembly.^30^ A study in *Xenopus tropicalis* shows that this p.(Lys7Gln) variant might interfere with the ability of WDR5 to localize to the bases of left-right organizer cilia, independent from the H3K4-methylation-related functions of WDR5.^31^ The p.(Lys7Gln) variant is located in a different region of the WDR5 protein compared with the variants here. Moreover, complete phenotypic details are not available for this individual, and it is currently unclear whether this reported individual has the *WDR5-*associated neurodevelopmental disorder presented in this study or this specific variant gives rise to a different disorder with different pathogenic mechanisms.

To the best of our knowledge, truncating variants (e.g., frameshift or nonsense variants) in *WDR5* have not been identified in any published disease cohort or in control individuals (e.g., in the gnomAD or TOPMED database). According to sequencing data from the gnomAD database, *WDR5* is extremely intolerant for both missense and loss-of-function variation. The gene has a loss-of-function observed/expected upper bound fraction (LOEUF) score of 0.124, which is well within the first decile of most highly constrained genes against loss of function.^18^ In contrast to the absence of truncating variants, heterozygous chromosomal microdeletions encompassing the whole *WDR5* gene have been reported; the Decipher database lists 11 heterozygous deletions that include *WDR5*.^32^ This means that haploinsufficiency for *WDR5* is compatible with life, but it is unclear how the loss of WDR5 contributes to specific phenotypes found in individuals with these deletions, as all deletions are larger than 3 Mb and encompass many other genes as well.

While our research provides clear evidence that rare *WDR5* variants can cause a Mendelian disorder, further studies are needed to assess the exact pathogenic mechanisms that play a role in causing the phenotypic features in individuals with this disorder. Our experimental approaches in *D. melanogaster* and human cell lines show intact mutant protein expression, localization, and protein-protein interactions for all variants tested. Three-dimensional protein structure analysis supports a model in which the variants disturb protein-protein interactions of WDR5 with COMPASS complex-related proteins. All in all, dominant-negative or gain-of-function mechanisms of pathogenicity might be most likely. Thus, future studies might benefit from testing for these possible effects instead of general loss-of-function effects. Also, as WDR5 is known to have many different functions in important cellular processes, future research on WDR5 should also target downstream consequences of impaired WDR5 functions, for example by using RNA expression analyses or histone methylation or DNA methylation profiling. In addition, the question remains whether all variants in our study exert pathogenicity via a similar mechanism or if different mechanisms are at play. Two individuals in our cohort (4 and 11) had distinct features compared with other individuals (severe micrognathia, small mouth, down-slanted palpebral fissures, and hearing loss), which might be caused by a distinct or additional mode of pathogenicity. Larger follow-up cohort studies are needed to perform detailed genotype-phenotype correlations for *WDR5* variants to carefully characterize the complete spectrum of *WDR5*-associated phenotypes and molecular underpinnings.

In conclusion, by identifying and characterizing individuals with rare *de novo* missense variants in *WDR5*, we suggest the presence of a novel Mendelian neurodevelopmental disorder. The associated phenotype consists of ID, speech and language impairments, epilepsy, and/or ASD. In addition, a wide spectrum of associated features is reported, including but not limited to aberrant growth parameters, skeletal abnormalities, and cardiac abnormalities. More clinical and functional studies are needed for a further delineation of the full clinical and mutational spectrum and the pathogenic mechanisms associated with this disorder by combining data from clinical and experimental approaches. Based on the results of our study, we can already add *WDR5* to the growing list of human disease genes encoding COMPASS complex family subunits, such as *KMT2A* (MIM: 159555), *KMT2B* (MIM: 606834), *KMT2C* (MIM: 606833), *KMT2D* (MIM: 602133), *KDM6A* (MIM: 300128), *SETD1A* (MIM: 611052), and *SETD1B* (MIM: 611055), thereby further confirming the important role of COMPASS complex family members, and more specifically WDR5, as highly important contributors to crucial (neuro)developmental processes.

## Data Availability

The clinical dataset used for this study is included in the supplemental information (Table S1). No other datasets were generated during this study.

## Supplemental information

Document S1. Supplemental materials and methods, Figures S1–S3, and Notes S1 and S2

Table S1. Clinical information and variant details of individuals with WDR5 variants

Document S2. Article plus supplemental information
