# Supplementary material for: A clustering of heterozygous missense variants in the crucial chromatin modifier WDR5 defines a new neurodevelopmental disorder
Source: HGG Adv. 2022 Nov 1;4(1):100157. doi: 10.1016/j.xhgg.2022.100157 (PMC9673101; doi:10.1016/j.xhgg.2022.100157)
Supplement: Document S1. Supplemental materials and methods, Figures S1–S3, and Notes S1 — and S2 [file mmc1.pdf]

HGGA, Volume *4*

Supplemental information

A clustering of heterozygous missense variants in the crucial chromatin modifier WDR5 defines a new neurodevelopmental disorder

Lot Snijders Blok, Jolijn Verseput, Dmitrijs Rots, Hanka Venselaar, A. Micheil Innes, Connie Stumpel, Katrin Õunap, Karit Reinson, Eleanor G. Seaby, Shane McKee, Barbara Burton, Katherine Kim, Johanna M. van Hagen, Quinten Waisfisz, Pascal Joset, Katharina Steindl, Anita Rauch, Dong Li, Elaine H. Zackai, Sarah E. Sheppard, Beth Keena, Hakon Hakonarson, Andreas Roos, Nicolai Kohlschmidt, Anna Cereda, Maria Iascone, Erika Rebessi, Kristin D. Kernohan, Philippe M. Campeau, Francisca Millan, Jesse A. Taylor, Hanns Lochmüller, Martin R. Higgs, Amalia Goula, Birgitta Bernhard, Danita J. Velasco, Andrew A. Schmanski, Zornitza Stark, Lyndon Gallacher, Lynn Pais, Paul C. Marcogliese, Shinya Yamamoto, Nicholas Raun, Taryn E. Jakub, Jamie M. Kramer, Joery den Hoed, Simon E. Fisher, Han G. Brunner, and Tjitske Kleefstra

Supplemental information

Supplemental Material & Methods

Study participants and consent

Individuals with *WDR5* variants were identified via matchmaking using GeneMatcher[1], the Dutch genetic diagnostic variant classification database (VKGL database)[2-4], ClinVar[5] and denovo-db[6]. Clinical data and details on variants were collected in a Castor EDC database[7]. Informed consent was obtained from all participating families. For all pictures of affected individuals, specific consent to publish clinical photographs was obtained. All procedures in this study matched local ethical guidelines of the participating centres, and are in accordance with the Declaration of Helsinki.

Next generation sequencing and *in silico* variant analyses

Details on next generation sequencing methods used to identify the *WDR5* variants found in all individuals are included in table S1. Variants were analysed using Alamut Visual 2.10. Conservation was studied using a Clustal[8] alignment of WDR5 amino acid sequences extracted from Uniprot (human, mouse and C. elegans)[9]. To assess the likelihood of pathogenicity, the prediction programs SIFT[10], PolyPhen-2[11] and CADD v1.4[12] were used.

Drosophila strains and RNAi knock down assays

Flies were reared on standard cornmeal-agar media at 25°C with a 12h/12h light/dark cycle at 70% humidity. The ubiquitous driver line, *Act5C-Gal4* (stock # 4414), mushroom body driver line, *R14H06-Gal4* (stock # 48667), *UAS-mCherry-RNAi* ($mCherry^{RNAi}$ stock # 35785), and *UAS-wds-RNAi* (wds^{RNAi} stock # 32952) were obtained from the Bloomington *Drosophila* stock center. Reference and variant *UAS-WDR5::HA* transgenic flies were generated as previously described[13]. Briefly, the Gateway (Thermo Fisher Scientific) compatible WDR5 cDNA open entry clone (NCBI Acc. #: BC001635.1) in pDONR223 was shuttled to the pGW-attB-HA[14] destination vector using LR Clonase II (Thermo Fisher Scientific, Cat# 11791020) as per manufacturer's protocol. To generate WDR5 variants, site-directed mutagenesis using the Q5 site-directed mutagenesis kit (NEB - E0554S) was employed. Constructs were verified using Sanger sequencing. All *UAS-WDR5::HA*

constructs were microinjected into embryos expressing φC31 integrase with a 2nd chromosome attP docking site VK37 (PBac{y[+]-attP}VK00037)[15] and identified by *w+* (encoded by the mini-white gene in pGW-attB-HA vector).

Wds RNAi lines were validated as previously described[16; 17]. Expression of *wds^{RNAi}* with a ubiquitous *Act-Gal4* driver resulted in complete lethality and qPCR on knockdown larvae showed that the expression level of *wds* is reduced 87% when compared to controls (p=0.0023, t-test).

**Primers for generation of UAS-WDR5 variant Drosophila<br>Forward and Reverse Primers (5' to 3')**	
UAS-WDR5::HA$^{p.A169P}$-F	GACTTTGCCACCTCACTCGGATC
UAS-WDR5::HA$^{p.A169P}$-R	TTGAGGCACTTCCCTGTT
WDR5::HA $^{p.R196C}$-F	TGGTCTCTGTTGCATCTGGGA
WDR5::HA $^{p.R196C}$-R	TCATAGCTACTTGAAACTATCAAG
WDR5::HA $^{p.A201V}$-F	TGGGACACCGTCTCGGGCCAG
WDR5::HA $^{p.A201V}$-R	GATGCGACAGAGACCATCATAGCTACTTGAAAC
WDR5::HA $^{p.T208M}$-F	TGCCTGAAGATGCTCATCGATGACGAC
WDR5::HA $^{p.T208M}$-R	CTGGCCCGAGGCGGTGTC
WDR5::HA $^{p.D213N}$-F	CATCGATGACAACAACCCCCC
WDR5::HA $^{p.D213N}$-R	AGCGTCTTCAGGCACTGG
WDR5::HA $^{p.K245R}$-F	GACTACAGCAGGGGGAAGTGC
WDR5::HA $^{p.K245R}$-R	CCAGAGCTTCAGAGTGTTG

Drosophila memory assays

Short-term memory (STM) and long-term memory (LTM) was assessed using courtship conditioning, as previously described[18-20]. Briefly, for each fly pair a courtship index (CI) was calculated, which is the proportion of time spent courting over 10 min. The memory index (MI) represents the percentage reduction in courtship behaviour in trained flies compared to naive and is used to compare memory between different genotypes. MI was calculated using the formula: $MI = (\bar{x}\ CI_{naïve} - \bar{x}\ CI_{trained}) / \bar{x}\ CI_{naïve}$. Statistics were generated as previously described[18; 20].

Western Blotting

For the western blot with lysates from *UAS-WDR5::HA* Drosophila, protein was extracted from 10 adult flies expressing *UAS-WDR5::HA* reference and variant transgenes via the ubiquitous *Act-Gal4* driver. Western blotting was preformed according to standard protocols using rabbit anti-HA (1:1000; Cell Signaling Technology C29F4), mouse anti-β-tubulin (1:5000; Developmental Studies Hybridoma Bank [DSHB]) primary antibodies, and horseradish-peroxidase-conjugated secondary antibodies goat anti-rabbit (1:3000; Bio-Rad 170-6515) and goat anti-mouse (1:3000; Bio-Rad 170-6516).

For the western blot with lysates from HEK293/T17 cells, whole-cell lysates were collected by treatment with RIPA buffer (Cell Signalling) supplemented with 1% PMSF and protease inhibitor cocktail (Roche). Samples were incubated for 20 min at 4 °C followed by centrifugation for 30 min at 12,000 rpm at 4 °C. Proteins were resolved on 4–15% Mini-PROTEAN TGX Precast Gels (Bio-Rad) and transferred onto polyvinylidene fluoride membranes using a TransBlot Turbo Blotting system (Bio-Rad). Membranes were blocked in 5% milk for 1 h at room temperature and then probed with mouse-anti-EGFP (1:8000; Clontech, 632380). Next, membranes were incubated with HRP-conjugated goat-anti-mouse IgG (1:10,000; Jackson ImmunoResearch) for 1 h at room temperature. Bands were visualized with Novex ECL Chemiluminescent Substrate Reagent (Invitrogen) using a ChemiDoc XRS + System (Bio-Rad). Equal protein loading was confirmed by probing with mouse-anti-β-actin antibody (1:10,000; Sigma, A5441).

DNA expression constructs for cell based assays and site-directed mutagenesis

WDR5 (NM_017588.3) and RbBP5 (NM_005057.4) coding sequences were amplified using primers listed below. cDNAs were subcloned using *HindIII/XbaI* (WDR5) and *SalI/BamHI* (RbBP5) restriction sites into pRluc and pYFP, created by modification of the pEGFP-C2 vector (Clontech) as described before.[21] Variants in WDR5 were generated using the QuikChange Lightning Site-Directed Mutagenesis Kit (Agilent). The primers used for site-directed mutagenesis are listed below.

**Primers for amplifying and subcloning of WDR5 and RbBP5 cDNAs**	
WDR5-cloning-F	AGAATTAAGCTTGTTGGCGACGGAGGAGAAGAA
WDR5-cloning-R	GGATCCTCTAGATTAGCAGTCACTCTTCCACA
RBBP5-cloning-F1	GACGATGTCGACTGCTGAACCTCGAGTTGCTGGA
RBBP5-cloning -R1	TCCGGTGGATCCTCATAACAGTTCTGAGATTG

**Primers used for site-directed mutagenesis**	
WDR5-R196C-R	CAAGTAGCTATGATGGTCTCTGTTGCATCTGGGACA
WDR5-R196C-F	TGTCCCAGATGCAACAGAGACCATCATAGCTACTTG
WDR5-A201V-R	ATCTGGGACACCGTCTCAGGCCAGTGC
WDR5-A201V-F	GCACTGGCCTGAGACGGTGTCCCAGAT
WDR5-T208M-R	AGGCCAGTGCCTGAAGATGCTCATCGATGAC
WDR5-T208M-F	GTCATCGATGAGCATCTTCAGGCACTGGCCT

Cell culture

HEK293T/17 cells (CRL-11268, ATCC) were cultured in DMEM supplemented with 10% fetal bovine serum and 1x penicillin-streptomycin (all Invitrogen) at 37°C with 5% CO_2. Transfections were performed using GeneJuice (Millipore) following the manufacturer's protocol.

Fluorescence microscopy

HEK293T/17 cells were grown on poly-D-lysine (Sigma) coated coverslips. Cells were fixed with 4% paraformaldehyde (PFA, Electron Microscopy Sciences) 48 h after transfection with YFP-tagged WDR5 variants. Nuclei were stained with Hoechst 33342 (Invitrogen). Fluorescence images were acquired with a Zeiss LSM880 confocal microscope and Airyscan detector, with a 2.0 zoom factor using ZEN Image Software (Zeiss).

Three-dimensional (3D) protein modelling

The effects of the identified variants on the WDR5 protein and its interaction with other proteins in the COMPASS family complexes were analyzed using YASARA View[22] with FoldX v4.0 plugin[23]. For the WDR5 structure, PDB file 2GNQ was used. PDB files 6KIV and 6KIW[24] were used for the analysis of the core COMPASS complexes, respectively; the 6UH5 file[25] of the yeast COMPASS model was used for the comparison with the human COMPASS complex. To optimize the position of amino-acid sidechains, all the PDB files that were used were corrected by the FoldX repair function using default settings. Different protein structures were aligned with SHEBA procedure[26], as implemented in YASARA.

BRET assay

BRET assays were performed as previously described [21]. HEK293T/17 cells were transfected in white clear-bottomed 96-well plates with increasing molar ratios of YFP-fusion proteins and constant amounts of Rluc-fusion proteins (donor/acceptor ratios of 1/0.5, 1/1, 1/2, 1/3, 1/6, 1/9). YFP and Rluc fused to a C-terminal nuclear localization signal were used as control proteins. After 48 h, medium was replaced with phenol red-free DMEM, supplemented with 10% fetal bovine serum (both Invitrogen), containing 60 µM EnduRen Live Cell Substrate (Promega). After incubation for 4 h at 37 °C, measurements were taken in live cells with an Infinite M200PRO Microplate reader (Tecan) using the Blue1 and Green1 filters. Corrected BRET ratios were calculated with the following formula: [Green1($_{\text{experimental condition}}$)/Blue1($_{\text{experimental condition}}$)] − [Green1($_{\text{control condition}}$)/Blue1($_{\text{control condition}}$)], with only the Rluc control protein expressed in the

control condition. Curve fitting was done with a non-linear regression equation assuming a single binding site (y = BRETmax * x / (BRET50 / x) using GraphPad Prism Software.

Figure S1: Immunoblot analysis with lysates from HEK293/T17 cells

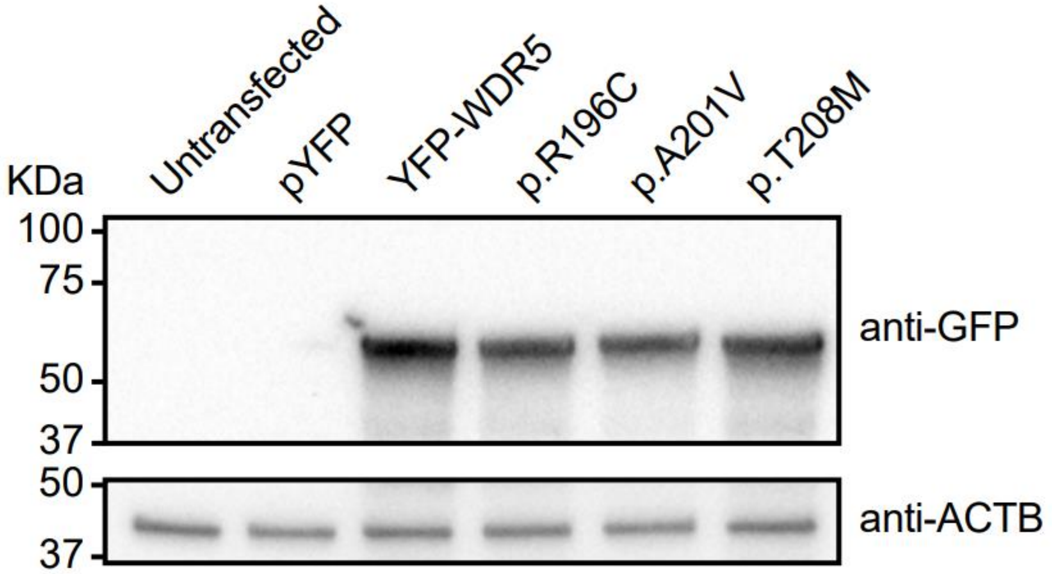

Immunoblot of whole-cell lysates (HEK293/T17 cells) expressing YFP-tagged WDR5 variants probed with anti-EGFP antibody. Expected molecular weight for all variants is ~65 kDa. The blot was stripped and probed for β-actin to ensure equal protein loading.

Figure S2: MetaDome intolerance visualization of WDR5

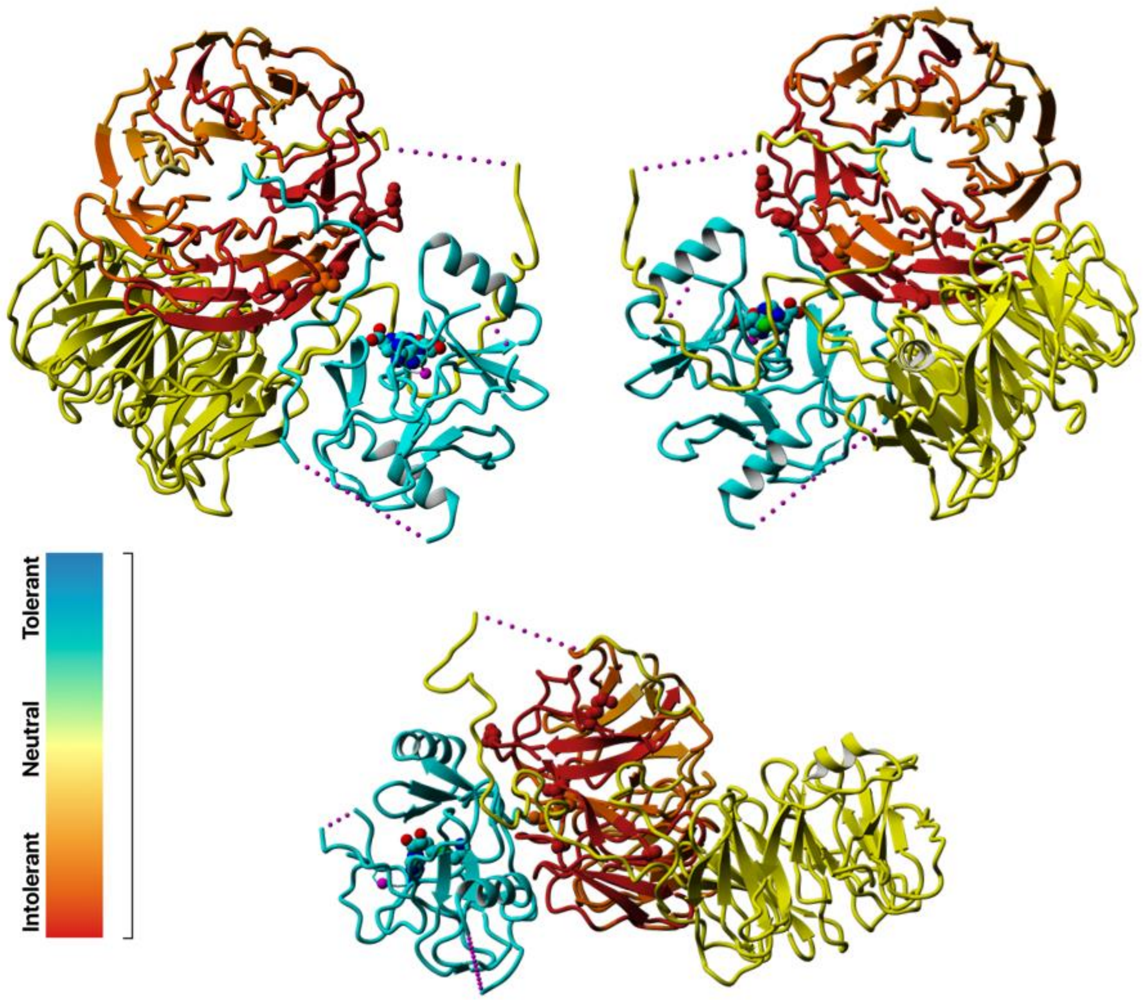

WDR5 is coloured in line with the MetaDome tolerance scale shown. RbBP5 is shown in yellow and KMT2A in cyan (PDB:6KIV). As can be seen in this figure, WDR5 is generally intolerant to missense variants, but WDR5 amino acids that are known to interact with other proteins are most intolerant (darker red).

Figure S3: Comparison of the core human KMT2A with the yeast COMPASS complexes

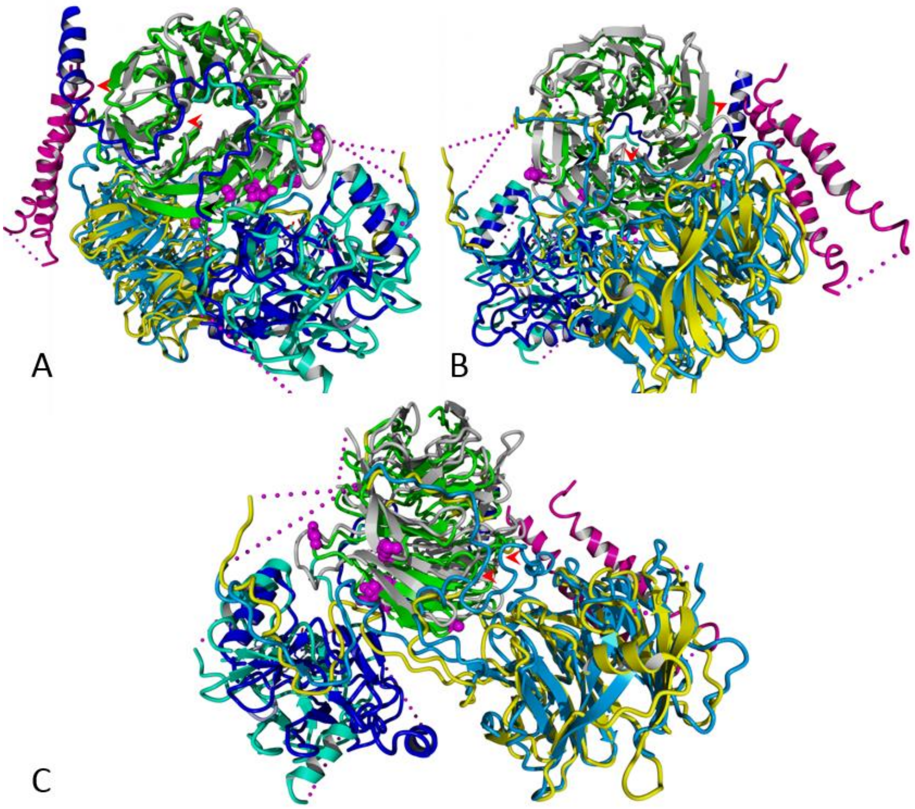

The alignment of human WDR5 in complex with RbBP5 and KMT2A from the core COMPASS complex (PDB:6KIV) with homologues of the yeast COMPASS complex (PDB:6UH5) is shown: WDR5 (green, p.33-332) with its homologue Swd3 (grey, p.16-326); RbBP5 (yellow, p.1-380) with its homologue Swd1 (light blue, p.1-435); KMT2A (cyan, p.3764-3969) with yeast homologue Set1c (dark blue, p.819-999). Additionally, yeast Spp1 (purple) is shown. The Spp1 homologue is not present in human COMPASS family complexes. The locations of the amino acids that are affected in patients identified in this study are shown with balls (magenta). Three different angles are shown: WDR5 faced from the WIN site (A), from the WBM site (B), and from the side between WIN and WBM (C).

The human core COMPASS/COMPASS family complexes (eg., KMT2A) are highly conserved and have a structure similar to the yeast COMPASS complex. Because the yeast COMPASS complex proteins in the 3D model are more complete, substantially more extensive interaction of the RbBP5 and KMT2A homologues with WDR5 homologues can be observed (red arrows). Additionally, another interaction site of the WDR5 homologue is observed with a Spp1 protein

These 3D modelling data, in addition to the high conservation level and low tolerance to the missense and LoF variants in the general population, suggest that also human WDR5 may have significantly more extensive interaction surfaces within COMPASS family complexes and other chromatin-remodelling complexes.

Supplemental note 1: Further details on individual 12 with c.742-2del (p.?) variant

Facial features of individual 12 with a *de novo* c.742-2del (p.?) variant

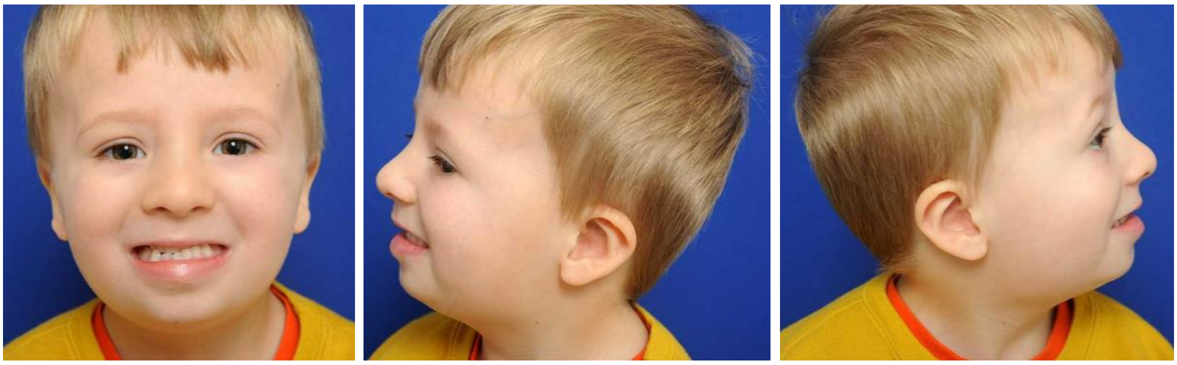

Facial photographs of individual 12 at age 4y1m

Variant analysis using splice prediction programs for c.742-2del (p.?) variant

Prediction program	Possible effect of c.742-2del variant
SpliceSiteFinder-like	Loss of acceptor site (exon 12), possible creation of alternative acceptor site 9bp upstream, resulting in in-frame deletion of 3AA (p.(Cys248_Lys250del)?
MaxEntScan	Loss of acceptor site (exon 12), possible creation of alternative acceptor site 9bp upstream, resulting in in-frame deletion of 3AA (p.(Cys248_Lys250del)?
GeneSplicer	Loss of acceptor site (exon 12), possible creation of alternative acceptor site 9bp upstream, resulting in in-frame deletion of 3AA (p.(Cys248_Lys250del)?
NNSPLICE	Loss of acceptor site (exon 12), skipping of exon 12?
SpliceAI	Loss of acceptor site (exon 12) resulting in skipping of exon 12 or creation of an alternative acceptor site 100bp downstream?

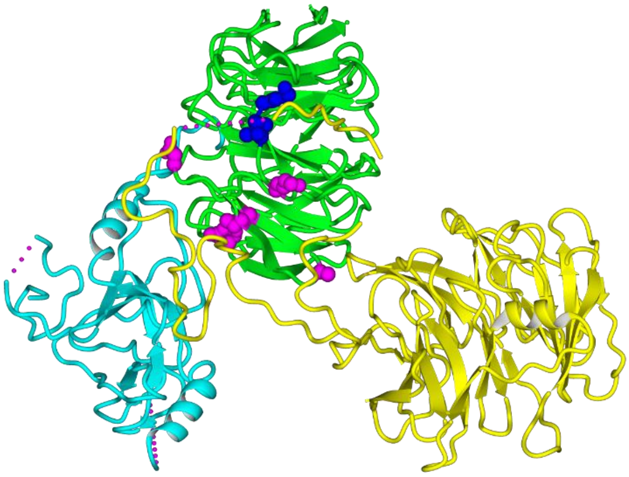

Three-dimensional analysis of c.742-2del variant and missense variants in WDR5

The WDR5 protein (green) in interaction with RbBP5 (yellow) and KMT2A/MLL1 (cyan) (PDB:6KIV). The location of amino acid substitutions (as the result of missense variants in our study) is shown in red. Amino acids involved in the possible in-frame deletion p.(Cys248_Lys250del) are depicted in dark blue.

Supplemental note 2: Detailed description and visualization of the predicted effect of identified WDR5 variants

p.(Ala169Pro)

Wild type residue role	Effect of the residue substitution
Ala169 is located in a turn from the third to fourth WDR5 beta-propeller. Despite the fact that the Ala169 is located in close proximity to the KMT2A, and KMT2C interaction sites, it does not directly interact with the KMT enzymes.	Change from the alanine to a larger proline at this position is predicted to result in a local backbone change, because of the rigid sidechain of the proline. This change is predicted to disturb the flexibility and local structure of WDR5, which will disrupt the binding to the KMT enzymes.

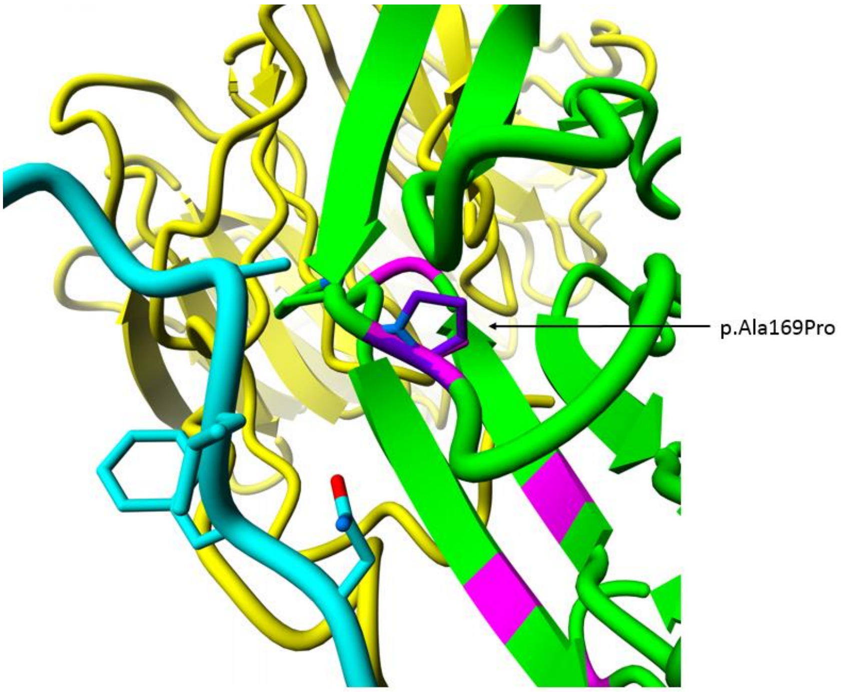

WDR5 (green) interaction with KMT2A (cyan) and RbBP5 (yellow), are shown from the core COMPASS complex (PDB:6KIV). The mutated aminoacid and nearby aminoacids are shown with sticks. The wild type alanine at the position p.169 is colored in magenta and the mutated proline in purple.

p.(Arg196Cys)

Wild type residue role	Effect of the residue substitution
Arg196 is located on the WDR5 lateral surface for interaction with RbBP5 and KMT2A enzymes. Arg196 interacts with Asn3779 in the KMT2A protein and Phe332 in the C-term tail of RbBBP5 but has no visible interactions with the KMT2C protein.	Cysteine is a much smaller residue and does not have a charge. Therefore, a change from the arginine to cysteine at this position would result in a loss of the hydrogen-bond with Asn3779 in KMT2A, as well resulting in an empty pocket between the WDR5, KMT2A and RbBBP5 interaction surfaces, which would lead to a loss of packing interactions and disruption of the interactions between the proteins.

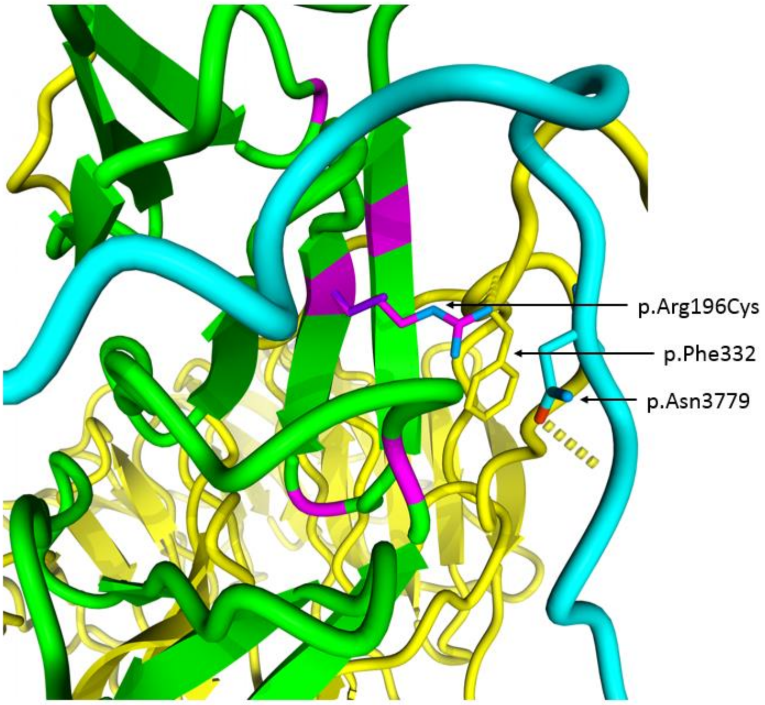

WDR5 (green) interaction with KMT2A (cyan) and RbBP5 (yellow), are shown from the core COMPASS complex (PDB:6KIV). The mutated aminoacid and nearby aminoacids are shown with sticks. The wild type arginine at the position p.196 is colored in magenta and the mutated cysteine in purple.

p.(Ala201Val)

Wild type residue role	Effect of the residue substitution
Ala201 is located on the WBM surface of WDR5 and interacts with RbBP5. It is located in clear proximity to Arg56 and Leu54 in the RbBP5 protein.	Despite the fact that valine is also small and non-polar, it has a bigger sidechain than alanine. Therefore, change to a valine at this position could affect the interaction with RbBP5 because of the change of the interaction surface.

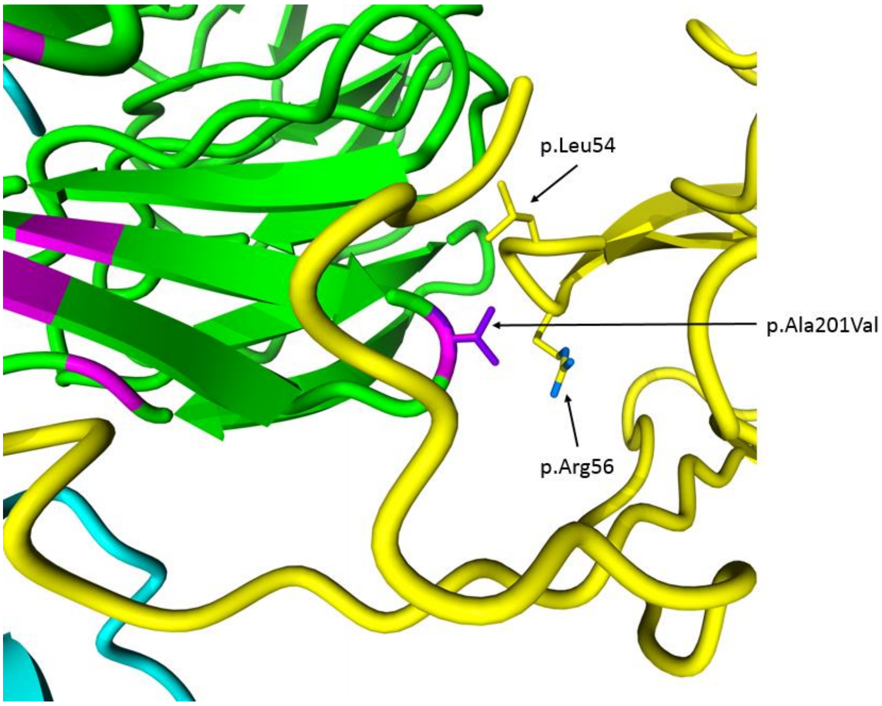

WDR5 (green) interaction with KMT2A (cyan) and RbBP5 (yellow), are shown from the core COMPASS complex (PDB:6KIV). The mutated aminoacid and nearby aminoacids are shown with sticks. The wild type alanine at the position p.201 is colored in magenta and the mutated valine in purple.

p.(Thr208Met)

Wild type residue role	Effect of the residue substitution
Thr208 in WDR5 interacts with several RbBP5 C-term tail amino acids (Ala331, Pro334). Additionally, it makes a hydrogen-bond with a backbone of Ala331 in RbBBP5.	Methionine has a substantially bigger size than threonine and is not able to form the hydrogen-bond with RbBP5 Ala331. Therefore, a change to methionine at this position is expected to disrupt the WDR5 interaction interface with RbBP5.

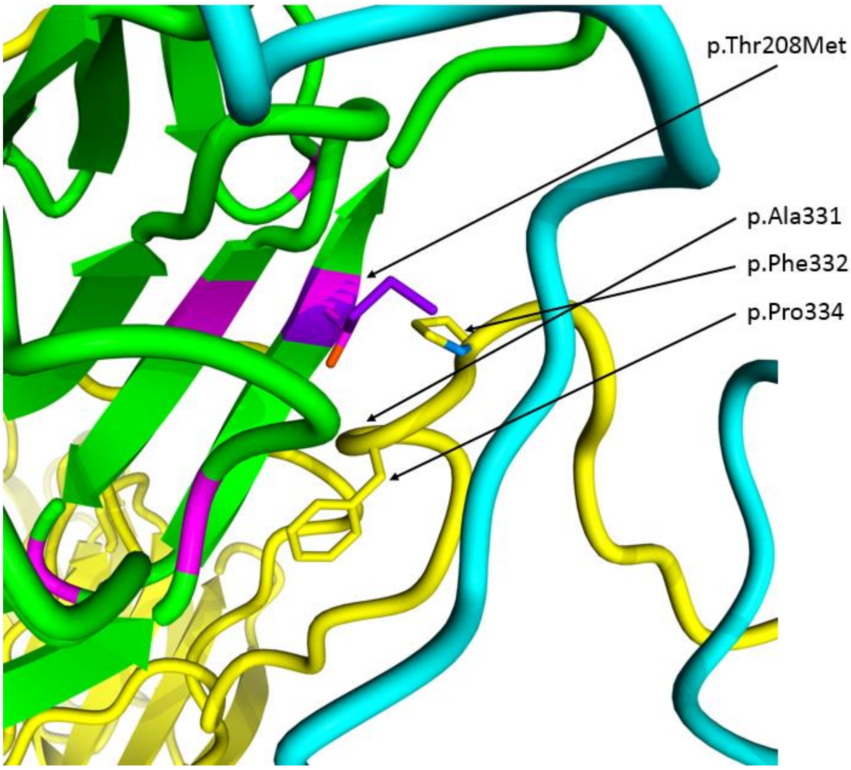

WDR5 (green) interaction with KMT2A (cyan) and RbBP5 (yellow), are shown from the core COMPASS complex (PDB:6KIV). The mutated aminoacid and nearby aminoacids are shown with sticks. The wild type threonine at the position p.208 is colored in magenta and the mutated methionine in purple.

p.(Asp213Asn)

Wild type residue role	Effect of the residue substitution
Asp213 is located in a WDR5 hydrophylic loop, which is involved in the interaction with the KMT enzymes. Although located distantly, it may interact with a positively charged KMT2A Lys3878, because the lysine has a highly flexible sidechain.<br><br>Additionally, Asp213 forms a hydrogen-bond with Asn235 in WDR5.	Change to the aspartate would result in a similar amino acid with similar size, although the negative charge of the aspartic acid would be lost. The hydrogen bond with WDR5 Asn235 would be lost due to this change, which may disrupt the stability and position of the loop, and, therefore, affect interaction with the KMT enzymes. Additionally, it can lose interactions with positively charged KMT residues. However, the exact effect of the variant is unknown.

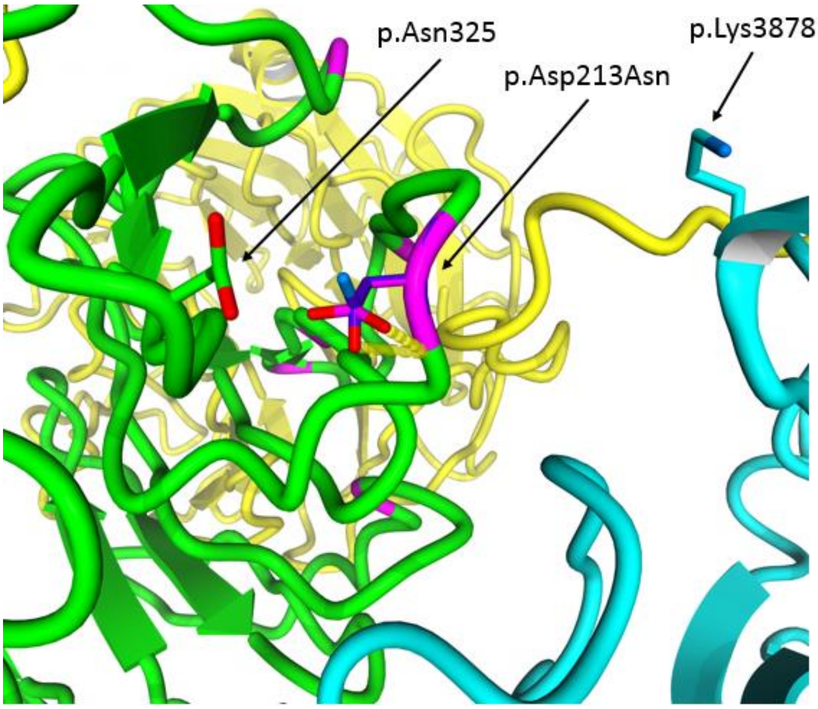

WDR5 (green) interaction with KMT2A (cyan) and RbBP5 (yellow) are shown from the core COMPASS complexes (PDB:6KIV and 6KIW, respectively). The mutated aminoacid and nearby aminoacids are shown with sticks. The wild type aspartic acid at the position p.213 is colored in magenta and the mutated aspartate in purple.

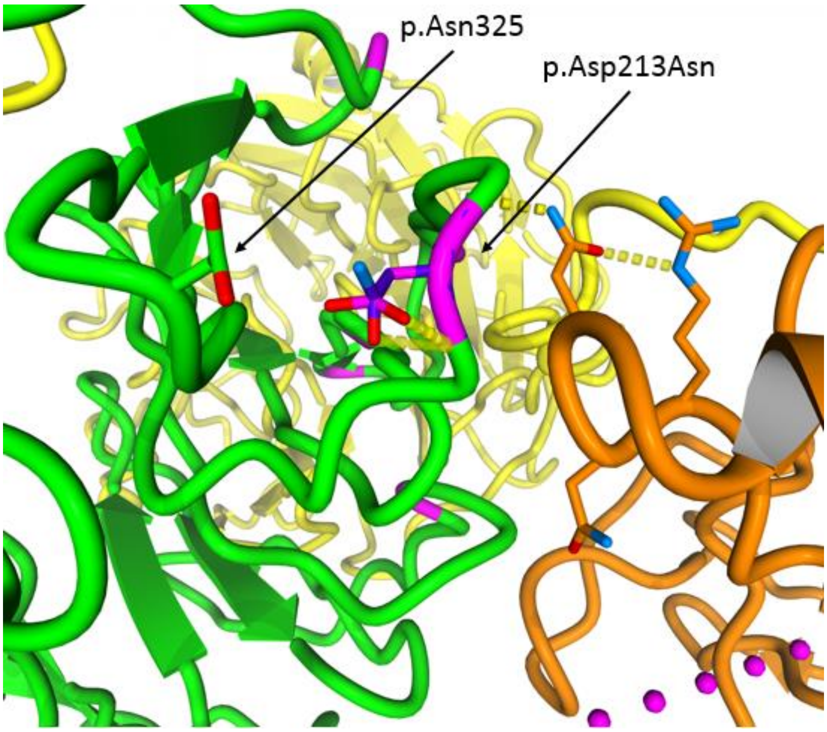

WDR5 (green) interaction with KMT2C (orange) and RbBP5 (yellow) are shown from the core COMPASS complexes (PDB:6KIV and 6KIW, respectively). The mutated aminoacid and nearby aminoacids are shown with sticks. The wild type aspartic acid at the position p.213 is colored in magenta and the mutated aspartate in purple.

p.(Lys245Arg)

Wild type residue role	Effect of the residue substitution
Lys245 located in a position that is a significant distance from the site of interaction with RbBP5 and KMT enzymes and is not known to be involved in a protein interaction.	A change from lysine to arginine at this position, would result in a similar amino acid by charge and flexibility of the side-chain with minimal effect on protein structure or interactions. Even though arginine is slightly larger, the effect of this variant is not clear.

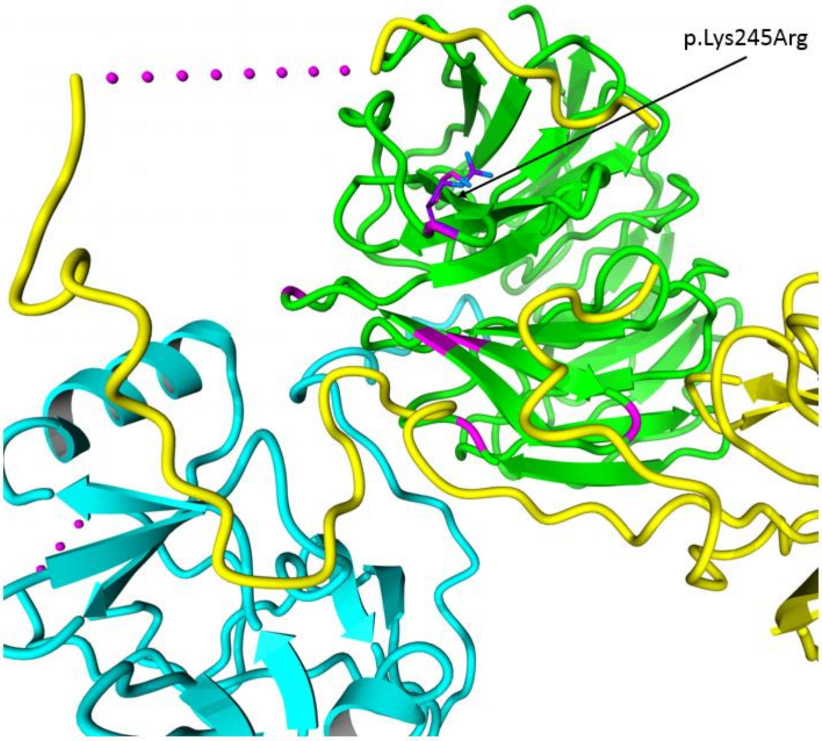

WDR5 (green) interaction with KMT2A (cyan) and RbBP5 (yellow), are shown from the core COMPASS complex (PDB:6KIV). The mutated aminoacid and nearby aminoacids are shown with sticks. The wild type lysine at the position p.245 is colored in magenta and the mutated arginine in purple.

Supplemental acknowledgements

Individuals 3 and 4 in this study were part of the DDD study cohort. The DDD study presents independent research commissioned by the Health Innovation Challenge Fund [grant number HICF-1009-003]. This study makes use of DECIPHER (http://decipher.sanger.ac.uk), which is funded by Wellcome. See Nature PMID: 25533962 or www.ddduk.org/access.html for full acknowledgement. Individual 6 was ascertained through the Care4Rare consortium. Individual 9 was part of the Undiagnosed Diseases Program Victoria (UDP-Vic). UDP-Vic acknowledges financial support from the Murdoch Children's Research Institute and the Harbig Foundation. The research conducted at the Murdoch Children's Research Institute was supported by the Victorian Government's Operational Infrastructure Support Program. Sequencing and analysis were provided by the Broad Institute of MIT and Harvard Center for Mendelian Genomics (Broad CMG) and was funded by the National Human Genome Research Institute, the National Eye Institute, and the National Heart, Lung and Blood Institute grant UM1 HG008900 and in part by National Human Genome Research Institute grant R01 HG009141.

References

1. Sobreira, N., Schiettecatte, F., Valle, D., and Hamosh, A. (2015). GeneMatcher: a matching tool for connecting investigators with an interest in the same gene. Hum Mutat 36, 928-930.
2. van der Velde, K.J., Imhann, F., Charbon, B., Pang, C., van Enckevort, D., Slofstra, M., Barbieri, R., Alberts, R., Hendriksen, D., Kelpin, F., et al. (2019). MOLGENIS research: advanced bioinformatics data software for non-bioinformaticians. Bioinformatics 35, 1076-1078.
3. Swertz, M.A., Dijkstra, M., Adamusiak, T., van der Velde, J.K., Kanterakis, A., Roos, E.T., Lops, J., Thorisson, G.A., Arends, D., Byelas, G., et al. (2010). The MOLGENIS toolkit: rapid prototyping of biosoftware at the push of a button. BMC Bioinformatics 11 Suppl 12, S12.
4. Swertz, M.A., and Jansen, R.C. (2007). Beyond standardization: dynamic software infrastructures for systems biology. Nat Rev Genet 8, 235-243.
5. Landrum, M.J., Lee, J.M., Benson, M., Brown, G.R., Chao, C., Chitipiralla, S., Gu, B., Hart, J., Hoffman, D., Jang, W., et al. (2018). ClinVar: improving access to variant interpretations and supporting evidence. Nucleic Acids Res 46, D1062-D1067.
6. Scholte, E., Van Duijn, E., Dijkxhoorn, Y., Noens, I., and Van Berckelaer-Onnes, I. (2008). Vineland screener 0–6 years: manual of the Dutch adaptation. PITS, Leiden.
7. Castor, E.D.C. (2019). Castor Electronic Data Capture. In. (
8. Sievers, F., Wilm, A., Dineen, D., Gibson, T.J., Karplus, K., Li, W., Lopez, R., McWilliam, H., Remmert, M., Söding, J., et al. (2011). Fast, scalable generation of high-quality protein multiple sequence alignments using Clustal Omega. In Mol Syst Biol. p 539.
9. The Uniprot Consortium (2018). UniProt: a worldwide hub of protein knowledge. Nucleic Acids Res 47, D506-D515.
10. Vaser, R., Adusumalli, S., Leng, S.N., Sikic, M., and Ng, P.C. (2016). SIFT missense predictions for genomes. Nat Protoc 11, 1-9.
11. Adzhubei, I.A., Schmidt, S., Peshkin, L., Ramensky, V.E., Gerasimova, A., Bork, P., Kondrashov, A.S., and Sunyaev, S.R. (2010). A method and server for predicting damaging missense mutations. Nat Methods 7, 248-249.
12. Kircher, M., Witten, D.M., Jain, P., O'Roak, B.J., Cooper, G.M., and Shendure, J. (2014). A general framework for estimating the relative pathogenicity of human genetic variants. Nature genetics 46, 310-315.
13. Harnish, J.M., Deal, S.L., Chao, H.T., Wangler, M.F., and Yamamoto, S. (2019). In Vivo Functional Study of Disease-associated Rare Human Variants Using Drosophila. J Vis Exp.
14. Bischof, J., Bjorklund, M., Furger, E., Schertel, C., Taipale, J., and Basler, K. (2013). A versatile platform for creating a comprehensive UAS-ORFeome library in Drosophila. Development 140, 2434-2442.
15. Venken, K.J., He, Y., Hoskins, R.A., and Bellen, H.J. (2006). P[acman]: a BAC transgenic platform for targeted insertion of large DNA fragments in D. melanogaster. Science 314, 1747-1751.
16. Mainland, R.L., Lyons, T.A., Ruth, M.M., and Kramer, J.M. (2017). Optimal RNA isolation method and primer design to detect gene knockdown by qPCR when validating Drosophila transgenic RNAi lines. BMC Res Notes 10, 647.
17. Chubak, M.C., Nixon, K.C.J., Stone, M.H., Raun, N., Rice, S.L., Sarikahya, M., Jones, S.G., Lyons, T.A., Jakub, T.E., Mainland, R.L.M., et al. (2019). Individual components of the SWI/SNF chromatin remodelling complex have distinct roles in memory neurons of the Drosophila mushroom body. Dis Model Mech 12.
18. Koemans, T.S., Oppitz, C., Donders, R.A.T., van Bokhoven, H., Schenck, A., Keleman, K., and Kramer, J.M. (2017). Drosophila Courtship Conditioning As a Measure of Learning and Memory. J Vis Exp.

19. Siegel, R.W., and Hall, J.C. (1979). Conditioned responses in courtship behavior of normal and mutant Drosophila. Proc Natl Acad Sci U S A 76, 3430-3434.
20. Kummeling, J., Stremmelaar, D.E., Raun, N., Reijnders, M.R.F., Willemsen, M.H., Ruiterkamp-Versteeg, M., Schepens, M., Man, C.C.O., Gilissen, C., Cho, M.T., et al. (2021). Characterization of SETD1A haploinsufficiency in humans and Drosophila defines a novel neurodevelopmental syndrome. Mol Psychiatry 26, 2013-2024.
21. Deriziotis, P., Graham, S.A., Estruch, S.B., and Fisher, S.E. (2014). Investigating protein-protein interactions in live cells using bioluminescence resonance energy transfer. J Vis Exp.
22. Krieger, E., and Vriend, G. (2014). YASARA View—molecular graphics for all devices—from smartphones to workstations. Bioinformatics 30, 2981-2982.
23. Schymkowitz, J., Borg, J., Stricher, F., Nys, R., Rousseau, F., and Serrano, L. (2005). The FoldX web server: an online force field. Nucleic acids research 33, W382-W388.
24. Xue, H., Yao, T., Cao, M., Zhu, G., Li, Y., Yuan, G., Chen, Y., Lei, M., and Huang, J. (2019). Structural basis of nucleosome recognition and modification by MLL methyltransferases. Nature 573, 445-449.
25. Hsu, P.L., Shi, H., Leonen, C., Kang, J., Chatterjee, C., and Zheng, N. (2019). Structural Basis of H2B Ubiquitination-Dependent H3K4 Methylation by COMPASS. Mol Cell 76, 712-723.e714.
26. Jung, J., and Lee, B. (2000). Protein structure alignment using environmental profiles. Protein Engineering, Design and Selection 13, 535-543.
