# Supplementary material for: A clustering of heterozygous missense variants in the crucial chromatin modifier WDR5 defines a new neurodevelopmental disorder
Source: HGG Adv. 2022 Nov 1;4(1):100157. doi: 10.1016/j.xhgg.2022.100157 (PMC9673101; doi:10.1016/j.xhgg.2022.100157)
Supplement: Document S2. Article plus supplemental information [file mmc3.pdf]

HGG Advances

REPORT

A clustering of heterozygous missense variants in the crucial chromatin modifier WDR5 defines a new neurodevelopmental disorder

Lot Snijders Blok,[1,2,3,44,45,*] Jolijn Verseput,[1,44] Dmitrijs Rots,[1,3] Hanka Venselaar,[4] A. Micheil Innes,[5] Connie Stumpel,[6] Katrin Õunap,[7,8] Karit Reinson,[7,8] Eleanor G. Seaby,[9,10] Shane McKee,[11] Barbara Burton,[12] Katherine Kim,[12] Johanna M. van Hagen,[13] Quinten Waisfisz,[13] Pascal Joset,[14] Katharina Steindl,[15] Anita Rauch,[15,16] Dong Li,[17,18,19] Elaine H. Zackai,[18,19] Sarah E. Sheppard,[17,18] Beth Keena,[18] Hakon Hakonarson,[17,19] Andreas Roos,[20,21,22] Nicolai Kohlschmidt,[23]

(Author list continued on next page)

Summary

WDR5 is a broadly studied, highly conserved key protein involved in a wide array of biological functions. Among these functions, WDR5 is a part of several protein complexes that affect gene regulation via post-translational modification of histones. We collected data from 11 unrelated individuals with six different rare *de novo* germline missense variants in *WDR5*; one identical variant was found in five individuals and another variant in two individuals. All individuals had neurodevelopmental disorders including speech/language delays (n = 11), intellectual disability (n = 9), epilepsy (n = 7), and autism spectrum disorder (n = 4). Additional phenotypic features included abnormal growth parameters (n = 7), heart anomalies (n = 2), and hearing loss (n = 2). Three-dimensional protein structures indicate that all the residues affected by these variants are located at the surface of one side of the WDR5 protein. It is predicted that five out of the six amino acid substitutions disrupt interactions of WDR5 with RbBP5 and/or KMT2A/C, as part of the COMPASS (complex proteins associated with Set1) family complexes. Our experimental approaches in *Drosophila melanogaster* and human cell lines show normal protein expression, localization, and protein-protein interactions for all tested variants. These results, together with the clustering of variants in a specific region of WDR5 and the absence of truncating variants so far, suggest that dominant-negative or gain-of-function mechanisms might be at play. All in all, we define a neurodevelopmental disorder associated with missense variants in *WDR5* and a broad range of features. This finding highlights the important role of genes encoding COMPASS family proteins in neurodevelopmental disorders.

WDR5 is a well-studied, highly conserved, and ubiquitously expressed protein[1–3] with impacts on many crucial developmental pathways as part of several different multiprotein complexes.[3,4] The indispensable function of WDR5 is illustrated by its high evolutionary conservation. Even simple multicellular organisms such as *Trichoplax adhaerens* have a protein with around 90% similarity to the 334 amino acids of the human ortholog.[1,3] Most of the protein complexes that WDR5 participates in affect gene regulation via post-translational modification of histones, e.g., the complex proteins associated with Set1 (COMPASS) family complexes,[5,6] the non-specific lethal (NSL) complex,[7] the Ada2a-containing (ATAC) complex,[8] and the nucleosome remodeling and deacetylase (NuRD) complex.[9] In addition to influencing cellular processes via protein-protein interactions, WDR5 is able to bind to >1,000 different endogenous RNA molecules.[10] WDR5 has an important role in embryonic stem cell (ESC) self-renewal and maintenance of a pluripotent state.[11,12] More recent studies have linked WDR5 to a newly discovered genetic compensation mechanism called nonsense-induced transcriptional compensation.[13] Moreover, WDR5 has been identified as a critical co-factor for retinoic acid signaling,[14] and directly interacts with p53 to regulate mouse ESC stem cell fate in a p53-dependent manner.[15] While the biological functions of the WDR5 protein have been studied from numerous angles, little is known about the impact of germline *WDR5* variants in humans.

[1]Human Genetics Department, Radboud University Medical Center, Nijmegen, the Netherlands; [2]Language & Genetics Department, Max Planck Institute for Psycholinguistics, Nijmegen, the Netherlands; [3]Donders Institute for Brain, Cognition and Behaviour, Radboud University Medical Center, Nijmegen, the Netherlands; [4]Centre for Molecular and Biomolecular Informatics, Radboud Institute for Molecular Life Sciences, Radboud University Medical Center, Nijmegen 6500HB, the Netherlands; [5]The Department of Medical Genetics and Alberta Children's Hospital Research Institute, Cumming School of Medicine, University of Calgary, Calgary, AB, Canada; [6]Department of Clinical Genetics and School for Oncology and Developmental Biology (GROW-School for Oncology and Reproduction), Maastricht UMC+, Maastricht, the Netherlands; [7]Department of Clinical Genetics, Genetics and Personalized Medicine Clinic, Tartu University Hospital, Tartu, Estonia; [8]Department of Clinical Genetics, Institute of Clinical Medicine, University of Tartu, Tartu, Estonia; [9]Translational Genomics Group, Broad Institute of MIT and Harvard, Cambridge, MA, USA; [10]Genomic Informatics Group, University Hospital Southampton, Southampton, UK; [11]Northern Ireland Regional Genetics Service, Belfast City Hospital, Belfast HSC Trust, Belfast BT9 7AB, UK; [12]Ann and Robert H. Lurie Children's Hospital and Northwestern University Feinberg School of Medicine, Chicago, IL, USA; [13]Department of Human Genetics, Amsterdam UMC, Vrije Universiteit Amsterdam, Amsterdam, the Netherlands; [14]Medical Genetics, Institute of Medical Genetics and Pathology, University Hospital Basel,

(Affiliations continued on next page)

© 2022 The Authors. This is an open access article under the CC BY license (http://creativecommons.org/licenses/by/4.0/).

Anna Cereda,[24] Maria Iascone,[25] Erika Rebessi,[26] Kristin D. Kernohan,[27] Philippe M. Campeau,[28,29] Francisca Millan,[30] Jesse A. Taylor,[31] Hanns Lochmüller,[32] Martin R. Higgs,[33] Amalia Goula,[33] Birgitta Bernhard,[34] Danita J. Velasco,[35] Andrew A. Schmanski,[35] Zornitza Stark,[36,37] Lyndon Gallacher,[36,37] Lynn Pais,[38] Paul C. Marcogliese,[39,40] Shinya Yamamoto,[39,40] Nicholas Raun,[41] Taryn E. Jakub,[42] Jamie M. Kramer,[41] Joery den Hoed,[2] Simon E. Fisher,[2,43] Han G. Brunner,[1,3,6] and Tjitske Kleefstra[1,3,*]

The initial finding of a *de novo* missense variant (c.623C>T, p.(Thr208Met)) in *WDR5* in a proband with childhood apraxia of speech[16] prompted us to investigate the effects and possible pathogenicity of rare germline variants in this gene. Using the GeneMatcher database[17] and other international collaborations, we collected clinical information on 11 unrelated individuals with rare *de novo* germline variants in *WDR5* that was collated from several clinical exome or genome sequencing studies (supplemental materials and methods). In these 11 individuals, six different missense variants were reported in *WDR5*: c.505C>G (p.(Ala169Pro)), c.586C>T (p.(Arg196Cys)), c.602C>T (p.(Ala201Val)), c.623C>T (p.(Thr208Met)), c.637G>A (p.(Asp213Asn)), and c.734A>G (p.(Lys245Arg)) (Table S1). All individuals had neurodevelopmental disorders with a spectrum of overlapping additional features (Figure 1; Table S1). Intellectual disability (ID) was present in 9/11 individuals, with a severity ranging from moderate ID (IQ 35–50, six individuals) to mild ID (IQ 50–70, three individuals). Speech delays were reported in all individuals, including nasal speech, developmental language disorders, verbal dyspraxia, and persistent stuttering. Three individuals remained nonverbal. All but one individual had delays in motor development, and hypotonia was reported in six individuals. Two individuals had ataxia. Seven individuals were diagnosed with different forms of epilepsy. Concerning the behavioral phenotype, four individuals had an autism spectrum disorder (ASD) diagnosis, and two individuals were diagnosed with attention deficit hyperactivity disorder (ADHD).

The individuals with *WDR5* variants showed divergent growth parameters (Figure 1). No clear correlation between height, weight, and head circumference was observed (Table S1), with the exception of two individuals (individuals 2 and 6) with a generalized overgrowth phenotype. Different abnormalities of the skeleton and limbs were present in a subset of individuals: scoliosis, kyphosis (with hemivertebra L5), bilateral clubfeet, and hemihypertrophy of one leg. In two individuals, heart abnormalities were reported: cardiac arrhythmias and decompensated heart failure requiring surgery in one individual, and left ventricular noncompaction cardiomyopathy in another individual. Four individuals were reported with frequent infections. However, one of these individuals had a combined immunodeficiency likely caused by a pathogenic missense variant in TNFRSF13B (Table S1). Overlapping facial features included a bulbous nasal tip, low-set, posteriorly rotated, and/or dysplastic ears, ptosis, and thin lip vermilion (Figure 2). Two individuals (4 and 11) had distinct facial features, with severe micrognathia (requiring tracheostomy in one), a small mouth, and prominent down-slanting palpebral fissures. Both had conductive hearing loss, too, a feature not reported in any of the other individuals. The variable expressivity of associated features and severity of symptoms is prominent, and we did not observe any clear genotype-phenotype correlation between specific variants and specific phenotypes. Even in five individuals

Basel, Switzerland; [15]Institute of Medical Genetics, University of Zuirch, Schlieren-Zurich, Switzerland; [16]University Children's Hospital Zurich, Zurich, Switzerland; [17]Center for Applied Genomics, The Children's Hospital of Philadelphia, Philadelphia, PA, USA; [18]Division of Human Genetics, Department of Pediatrics, The Children's Hospital of Philadelphia, Philadelphia, PA, USA; [19]Department of Pediatrics, Perelman School of Medicine at the University of Pennsylvania, Philadelphia, PA, USA; [20]Department of Neuropediatrics, Developmental Neurology and Social Pediatrics, Centre for Neuromuscular Disorders in Children, University Hospital Essen, University of Duisburg-Essen, Essen, Germany; [21]Children's Hospital of Eastern Ontario Research Institute, Ottawa, ON, Canada; [22]Department of Neurology, University Hospital Bergmannsheil, Heimer Institute for Muscle Research, 44789 Bochum, Germany; [23]Institute of Clinical Genetics and Tumor Genetics, Bonn, Germany; [24]Department of Pediatrics, ASST Papa Giovanni XXIII, Bergamo, Italy; [25]Laboratory of Medical Genetics, ASST Papa Giovanni XXIII, Bergamo, Italy; [26]Pediatric Neurological Unit and Epilespy Center, Fatebenefratelli Hospital, Milan, Italy; [27]Newborn Screening Ontario, Children's Hospital of Eastern Ontario and Children's Hospital of Eastern Ontario Research Institute, Ottawa, ON, Canada; [28]CHU Sainte-Justine Research Center, Montreal, QC H3T 1C5, Canada; [29]Sainte-Justine Hospital, University of Montreal, Montreal, QC H3T 1C5, Canada; [30]GeneDx, Gaithersburg, MD 20877, USA; [31]Division of Plastic Surgery, Department of Surgery, The Children's Hospital of Philadelphia, Philadelphia, PA, USA; [32]Children's Hospital of Eastern Ontario Research Institute, Division of Neurology, Department of Medicine, the Ottawa Hospital, Brain and Mind Research Institute, University of Ottawa, Ottawa, Canada; [33]Lysine Methylation and DNA Damage Laboratory, Institute of Cancer and Genomic Sciences, University of Birmingham, Birmingham, UK; [34]North-West Thames Regional Genetics Service, North West London Hospitals NHS Trust, Munroe-Meyer, Harrow, UK; [35]Institute for Genetics and Rehabilitation, University of Nebraska Medical Center, Omaha, NE, USA; [36]Victorian Clinical Genetics Services, Murdoch Children's Research Institute, Melbourne, VIC, Australia; [37]Department of Paediatrics, University of Melbourne, Melbourne, VIC, Australia; [38]Broad Center for Mendelian Genomics, Program in Medical and Population Genetics, Broad Institute of MIT and Harvard, Cambridge, MA, USA; [39]Department of Molecular and Human Genetics, Baylor College of Medicine, Houston, TX, USA; [40]Jan and Dan Duncan Neurological Research Institute, Texas Children's Hospital, Houston, TX 77030, USA; [41]Department of Biochemistry and Molecular Biology, Faculty of Medicine, Dalhousie University, Halifax, NS, Canada; [42]International Max Planck Research School for Language Sciences, Max Planck Institute for Psycholinguistics, Nijmegen, the Netherlands; [43]Donders Institute for Brain, Cognition & Behaviour, Radboud University, Nijmegen, the Netherlands
[44]These authors contributed equally.
[45]Lead contact
*Correspondence: lot.snijdersblok@radboudumc.nl (L.S.B.), tjitske.kleefstra@radboudumc.nl (T.K.)
https://doi.org/10.1016/j.xhgg.2022.100157.

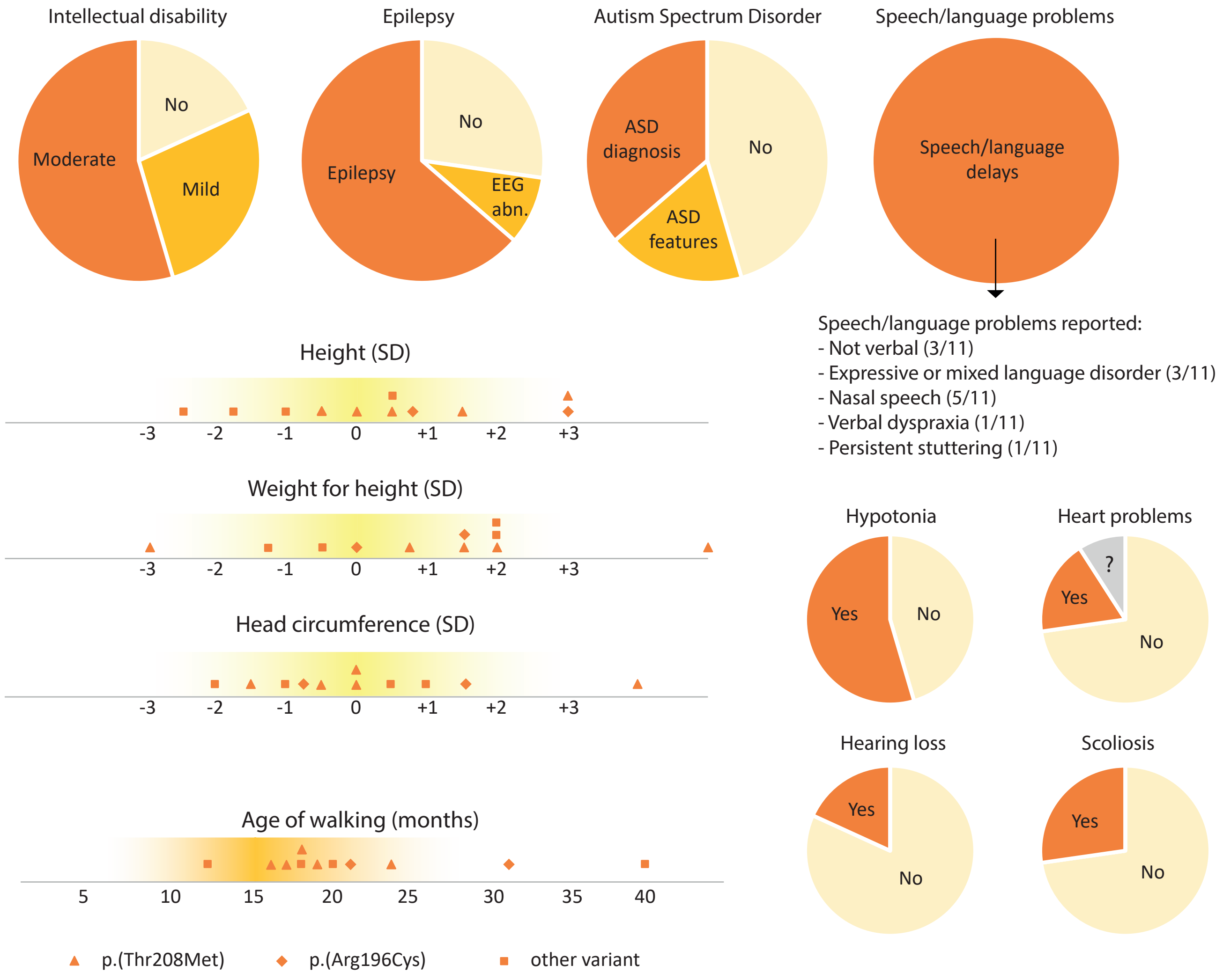

Figure 1. Clinical features reported in individuals with *WDR5* variants
Graphical overview of clinical features reported in 11 individuals with *WDR5* missense variants. Growth parameters are shown as standard deviations to the mean for a certain age. All graphs include data for 11 individuals (N = 11). EEG abn., EEG abnormalities. A more detailed overview of clinical features can be found in Table S1.

with the exact same missense variant, p.(Thr208Met), a different clinical presentation was seen, e.g., borderline versus moderate ID and normal growth parameters versus a generalized overgrowth phenotype. Clinical features reported in individuals in our cohort are described in more detail in Table S1.

Regarding the molecular aspects of *WDR5*, we found six different missense variants in 11 unrelated individuals: the p.(Thr208Met) variant was reported in five individuals and the p.(Arg196Cys) in two individuals. All variants were confirmed to be *de novo*, and none were in the gnomAD database,[18] showing that these variants are extremely rare on a population level. We used *in silico* prediction programs to evaluate pathogenicity for the variants, and while all CADD scores were above 22, SIFT and PolyPhen-2 predicted only three to be pathogenic: p.(Ala169Pro), p.(Arg196Cys), and p.(Thr208Met) (Table S1). Using a linear model of WDR5, we found all missense variants to be located within or flanking the fourth and the fifth WD40 domain of WDR5 (Figure 3A). As a member of the WD40 repeat protein family, WDR5 has seven WD40 domains that each form a propeller-like wing[19] of the final "barrel" -shaped protein (Figure 3B).

We used different experimental approaches in fruit flies (*Drosophila melanogaster*) and human cell lines to assess possible pathogenic effects of *WDR5* variants. Previous studies showed that the histone methyltransferase subunits of the COMPASS complexes are required in *Drosophila* memory neurons of the mushroom body for normal memory function.[20,21] Here, we used the same approach to test mushroom-body specific knockdown of the fly WDR5-ortholog *will die slowly* (*wds*) (supplemental materials and methods). While *wds* was efficiently knocked down in the transgenic RNAi line (wds^{RNAi}) that we used, we did not observe any differences in short- or long-term memory outcomes upon *wds* knockdown compared with controls (mCherry^{RNAi}) (Figure 3C). This suggests that

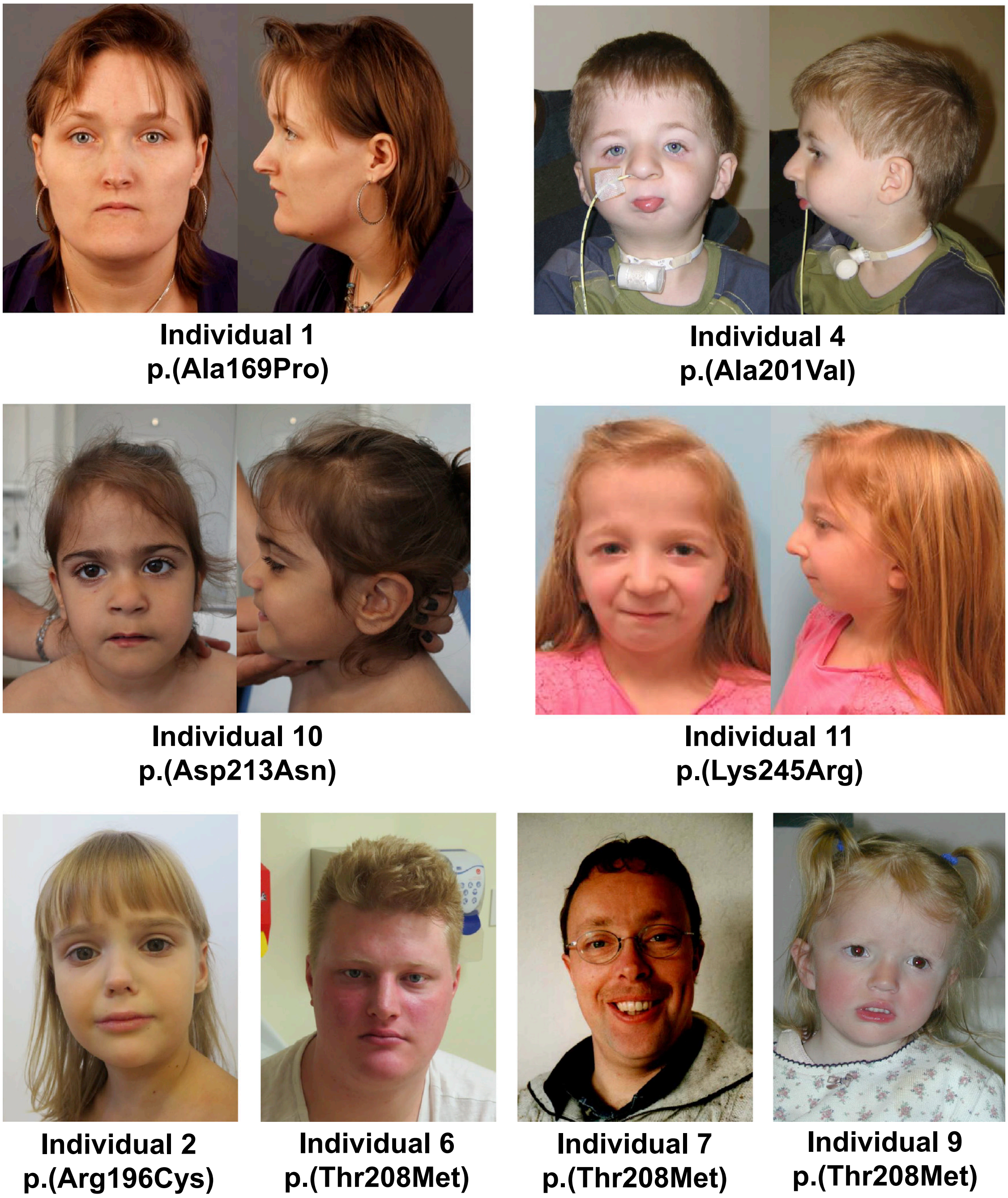

Figure 2. Facial features in individuals with six different *WDR5* variants
Facial images of eight individuals with a heterozygous *WDR5* variant. Several overlapping facial features are seen, such as a bulbous nasal tip (individuals 2, 4, and 10), low-set, posteriorly rotated, and/or dysplastic ears (individuals 2, 4, 7, and 10), ptosis (individual 11), and thin upper lip vermilion (individuals 4, 10, and 11). In addition, individuals 4 and 11 have severe micrognathia, a small mouth, and down-slanting palpebral fissures.

memory neurons are more resilient to loss of *wds/WDR5* than to loss of COMPASS complex enzymatic subunits. In addition, we assessed the expression of reference or variant human WDR5 proteins tagged with C′-3xHA tag (WDR:HA) driven with a ubiquitous driver in transgenic *Drosophila* strains using western blot (supplemental materials and methods). No difference was observed for the protein expression levels of any of the six examined WDR5 proteins with amino acid substitutions (p.(Ala169Pro), p.(Arg196Cys), p.(Ala201Val), p.(Thr208Met), p.(Asp213 Asn), and p.(Lys245Arg)) compared with wild-type WDR5 (Figure 3D). Consistently, three WDR5 variant proteins (p.(Arg196Cys), p.(Ala201Val), and p.(Thr208Met)) did not show any differences in subcellular localization (Figure 3E) or protein expression levels (Figure S1) compared with wild-type WDR5 when overexpressed as yellow fluorescent protein (YFP)-fusion proteins in HEK293/T17 cells (supplemental materials and methods).

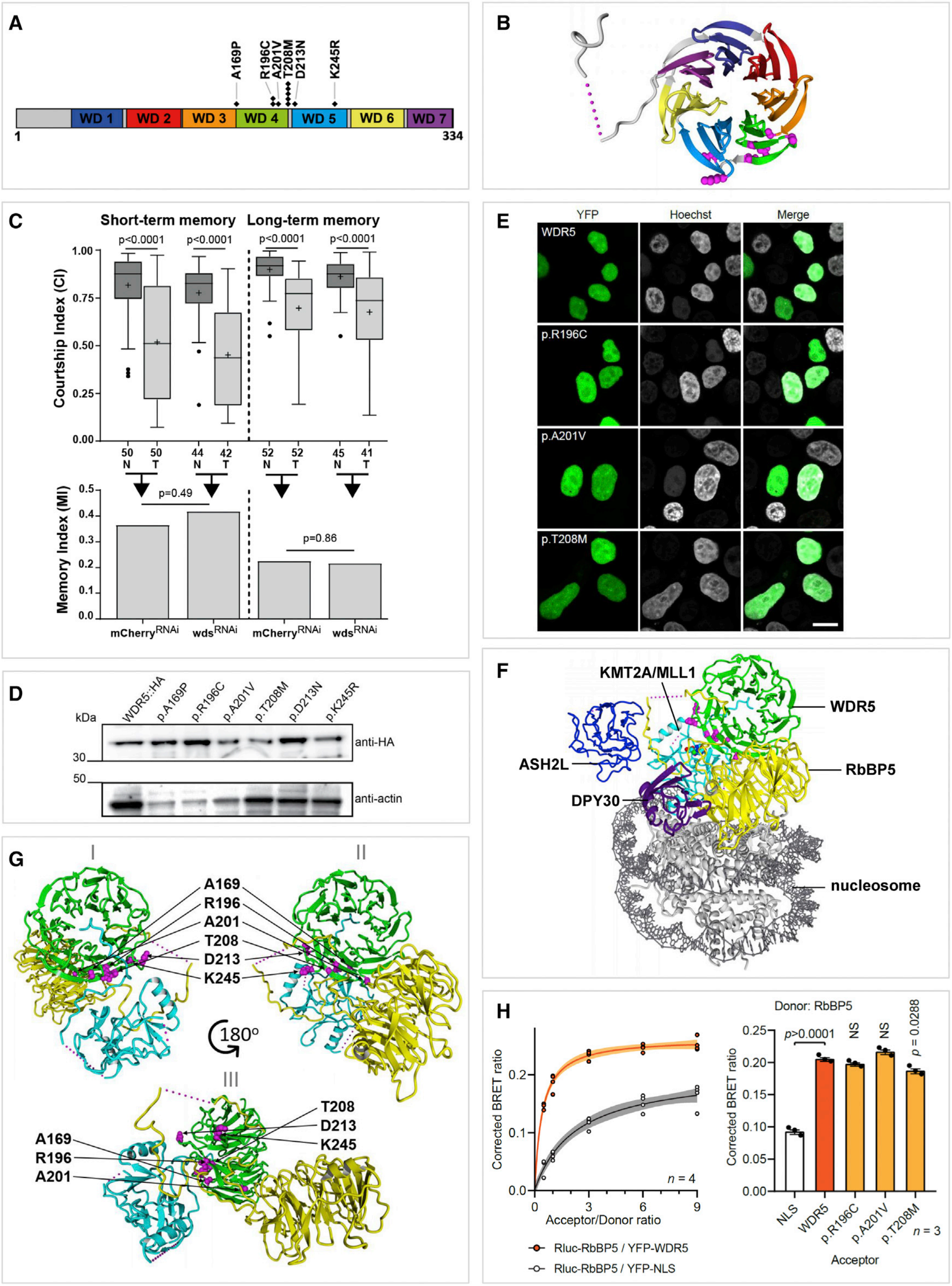

Figure 3. ***In silico, in vitro,*** **and** ***in vivo*** **studies of the effect of** ***WDR5*** **missense variants**

(A) Linear structure of WDR5 protein (334 amino acids) with the seven different WD40 domains and all identified missense variants shown; in total, six different missense variants were found in 11 individuals: one variant (p.(Thr208Met)) was found in five unrelated individuals and another variant (p.(Arg196Cys)) in two individuals.

(B) Three-dimensional visualization of WDR5 (PDB: 2GNQ); locations of the amino acids involved in missense variants are shown with magenta balls. Colors of the different domains match with the colors used in (A).

(C) Courtship memory was assessed in mushroom-body-specific wds RNAi knockdown flies (wds^{RNAi}) compared with controls expressing an RNAi against mCherry (mCherry^{RNAi}). Boxplots show the distribution of courtship indices (CIs) for naive (N) and trained (T) flies (top panel). Memory was observed when a significant reduction in CI occurred between naive and trained conditions (Mann-Whitney test). + indicates the mean. N is indicated along the x axis. Bar graphs show the memory indices (MIs), which are single values derived

(legend continued on next page)

Taken together, both *in vivo* and *in vitro* studies showed that the missense variants functionally assessed in our study lead to stable and normally expressed WDR5 proteins. This, combined with the absence of truncating variants in our cohort, argues against a sole loss-of-function effect as the underlying pathogenic mechanism. The fact that all variants found in affected individuals are missense variants and the clustering and recurrence of these variants at specific positions suggest that other mechanisms might be at play, such as dominant-negative or gain-of-function effects.

Through three-dimensional protein structure analysis, we determined that the amino acid residues affected by the six missense variants cluster together in the three-dimensional protein structure of WDR5, and more specifically on the surface of one side of the encoded protein. An interesting hypothesis for pathogenicity of the missense variants is that the amino acid substitutions disrupt specific interactions with other proteins. A landscape of intolerance for genetic variation in the *WDR5* gene visualized in the three-dimensional structure of the encoded protein shows that while WDR5 is generally intolerant to missense variants, residues that interact with other proteins have highest intolerance for normal variation (Figure S2). WDR5 is able to act as a molecular adapter to facilitate protein-protein interactions[3] using two distinct binding sites identified in previously performed co-precipitation experiments: the "WDR5-interacting" (WIN) site[22-24] and the "WDR5-binding motif" (WBM) site[24,25] located on opposite sides of the protein. The missense variants in our study were not located in the vicinity of these two most well-studied binding locations. However, recently published cryo-electron microscopy three-dimensional structures of the COMPASS complexes revealed a region, located between the WIN and WBM binding sites, that is involved in the interaction with RbBP5 and histone-lysine methylase (KMT) enzymes in these complexes.[26] Five out of the six missense variants in our cohort map within this RbBP5/KMT interaction region. Based on the three-dimensional structure analysis of the COMPASS complexes, p.(Ala169Pro) and p.(Asp213Asn) are predicted to affect the WDR5 interaction with KMT enzymes, and p.(Ala201Val) and p.(Thr208Met) are predicted to affect the interaction with the RbBP5 enzymes, while p.(Arg196Cys) most likely influences the interaction with both enzymes (Figure 3G). The effects of the p.(Lys245Arg) variant cannot be predicted using the currently available three-dimensional structures. A detailed description of the predicted effects of all variants, from the perspective of the structural modeling analyses, is provided in Note S2.

WDR5 is a crucial core protein within the COMPASS complex family: it is essential for complex assembly and activity.[27,28] In this context, it is important to note that the detailed three-dimensional structures used for these analyses are unfortunately only available for the COMPASS complex and not for all other complexes and interactions in which WDR5 is involved. Therefore, it remains unclear whether the predicted disruptive effects on WDR5 interactions are specific to those with RbBP5/KMT2 or if interactions with other molecules might also be disturbed. Based on three-dimensional protein structure analysis of COMPASS complexes, it seems that differently composed COMPASS complexes make use of different interaction surfaces of WDR5. Some variants might therefore disrupt interactions in only one specific complex. As WDR5 seems to act as an "adapter" protein, forming links between different molecules, disruption of protein-protein interactions within the complex might have important effects on complex activity.

As a functional follow up, we used bioluminescence resonance energy transfer (BRET) assays in live cells and were able to confirm the WDR5-RbBP5 interaction (Figure 3H). However, we could not demonstrate a biologically significant disruption of the interaction between WDR5 missense variants (p.(Arg196Cys), p.(Ala201Val), and p.(Thr208Met)) and RbBP5 (Figure 3H). While this result does not support the findings of our protein structure analyses, which predict a disruption of the interaction with one of the COMPASS family protein complex members, it does not exclude possible effects of missense variants on the formation and composition of the COMPASS complex or an altered activity of the complex due to the

from the above CIs (indicated by arrows) according to the formula: $MI = (\overline{X}\ CI_{naive} - \overline{X}\ CI_{trained}) / \overline{X}\ CI_{naive}$. MIs were consistent between controls and wds^{RNAi} lines (randomization test, 10,000 bootstrap replicates).

(D) Detection of WDR5 reference and variant proteins in adult flies by western blot. Representative bands for HA-tagged WDR5 reference and variant proteins at 36.6 kDa (top) along with the actin loading control 41 kDa (bottom). *UAS-WDR5::HA* reference and variant transgenes were expressed ubiquitously using *Actin-Gal4*.

(E) Direct fluorescence micrographs of nuclei of HEK293T/17 cells expressing YFP-WDR5 fusion proteins (green). Nuclei were stained with Hoechst 33342 (blue). Scale bar: 10 μm.

(F) WDR5 (green) is shown as part of the core COMPASS complex, with RbBP5 (yellow), ASH2L (blue), DPY30 (purple), and KMT2A (cyan) (PDB: 6KIV). The nucleosome is shown in gray. The locations of affected amino acids in individuals with missense variants are shown with magenta balls.

(G) WDR5 (green; p.33–332) is shown together with RbBP5 (yellow; p.1–380) and KMT2A (cyan; p.3764–3969) as part of the core COMPASS complex (PDB: 6KIV). The locations of affected amino acids in individuals with missense variants are shown with magenta balls from three different angles facing the WIN site (I), the WBM site (II), and a side between WIN and WBM (III).

(H) BRET assays for WDR5-RbBP5 interaction in live cells. Left, mean BRET saturation curves ±95% confidence interval fitted using a nonlinear regression equation assuming a single binding site (n = 4; y = BRETmax * x/(BRET50/x); GraphPad) showing a strong BRET signal for Rluc-RbBP5 with YFP-WDR5. Right, corrected BRET values measured with an acceptor/donor ratio of 1:1 (n = 3, one-way ANOVA and post-hoc Bonferroni test). NLS, YFP fused to a C-terminal nuclear localization signal as control protein. p values show significance for the comparison of WDR5 variant with the WDR5 wild-type.

missense variants. In our BRET assays, we investigated a single protein-protein interaction of heterologously expressed fluorescently tagged proteins, and we may not have been able to detect subtle shifts in WDR5-RbBP5 interaction dynamics or possible changes in the composition of the protein complexes involved.

Using three-dimensional structure analyses, we were not able to predict a likely pathogenic mechanism for the p.(Lys245Arg) variant. One hypothesis to explain pathogenicity of this variant could be that the variant affects a so-far-uncharacterized interaction site with RbBP5 or KMT2A/C, as a comparison of available three-dimensional structures between human KMT2A and yeast COMPASS complex suggests even more extensive interaction surfaces between WDR5 and histone methylases (Figure S3). Another hypothesis is that the p.(Lys245Arg) variant affects the interaction with other molecules that are not involved in the COMPASS complex.

In addition to the 11 individuals with missense variants in our study, we identified a *de novo* intronic variant in *WDR5* affecting a canonical splice site (c.742-2del) in an individual with multiple skeletal abnormalities, a cleft palate, acquired microcephaly, short stature (−2 SD), and normal development at 4 years of age (Table S1; Note S1). The skeletal abnormalities included right radial hypoplasia, absent right thumb, four metacarpals of the left hand, hypoplastic thumb bones, soft tissue syndactyly 1-2 of the left hand, and T7 butterfly vertebra with normal lower extremities. Of note, this patient also had a left ventricular noncompaction cardiomyopathy, a rare cardiac abnormality also present in individual 10, with missense variant p.(Asp231Asn). While five different *in silico* splice prediction tools all predicted a loss of the acceptor site of the 12th exon of WDR5, the consequences at mRNA and protein levels are unclear. Three of the splice prediction tools predicted the creation of a new acceptor site 9 bp upstream from the current acceptor site, resulting in an in-frame loss of three amino acids (p.248–250) (Note S1). Interestingly, these three amino acids are located on the surface of WDR5 in the region that interacts with RbBP5. All in all, it is likely that this *de novo* variant affecting a canonical splice site is a pathogenic variant, but it remains unclear how the effect of this splice variant relates to the effect of the missense variants reported in our study.

Our study represents the characterization of multiple probands with a Mendelian disorder associated with germline variants in *WDR5*. It is worth mentioning that, beyond the cases described here, one additional *de novo* variant in *WDR5* has been reported in the literature: a p.(Lys7Gln) variant, found in a child with a conotruncal heart defect with a right aortic arch.[29] This missense variant is located in the N-terminal tail of WDR5, an intrinsically disordered region of the protein (not available for three-dimensional protein structure analysis), which is not involved in the beta-propeller structure of WDR5, and has been shown to be dispensable for COMPASS complex assembly.[30] A study in *Xenopus tropicalis* shows that this p.(Lys7Gln) variant might interfere with the ability of WDR5 to localize to the bases of left-right organizer cilia, independent from the H3K4-methylation-related functions of WDR5.[31] The p.(Lys7Gln) variant is located in a different region of the WDR5 protein compared with the variants here. Moreover, complete phenotypic details are not available for this individual, and it is currently unclear whether this reported individual has the *WDR5*-associated neurodevelopmental disorder presented in this study or this specific variant gives rise to a different disorder with different pathogenic mechanisms.

To the best of our knowledge, truncating variants (e.g., frameshift or nonsense variants) in *WDR5* have not been identified in any published disease cohort or in control individuals (e.g., in the gnomAD or TOPMED database). According to sequencing data from the gnomAD database, *WDR5* is extremely intolerant for both missense and loss-of-function variation. The gene has a loss-of-function observed/expected upper bound fraction (LOEUF) score of 0.124, which is well within the first decile of most highly constrained genes against loss of function.[18] In contrast to the absence of truncating variants, heterozygous chromosomal microdeletions encompassing the whole *WDR5* gene have been reported; the Decipher database lists 11 heterozygous deletions that include *WDR5*.[32] This means that haploinsufficiency for *WDR5* is compatible with life, but it is unclear how the loss of WDR5 contributes to specific phenotypes found in individuals with these deletions, as all deletions are larger than 3 Mb and encompass many other genes as well.

While our research provides clear evidence that rare *WDR5* variants can cause a Mendelian disorder, further studies are needed to assess the exact pathogenic mechanisms that play a role in causing the phenotypic features in individuals with this disorder. Our experimental approaches in *D. melanogaster* and human cell lines show intact mutant protein expression, localization, and protein-protein interactions for all variants tested. Three-dimensional protein structure analysis supports a model in which the variants disturb protein-protein interactions of WDR5 with COMPASS complex-related proteins. All in all, dominant-negative or gain-of-function mechanisms of pathogenicity might be most likely. Thus, future studies might benefit from testing for these possible effects instead of general loss-of-function effects. Also, as WDR5 is known to have many different functions in important cellular processes, future research on WDR5 should also target downstream consequences of impaired WDR5 functions, for example by using RNA expression analyses or histone methylation or DNA methylation profiling. In addition, the question remains whether all variants in our study exert pathogenicity via a similar mechanism or if different mechanisms are at play. Two individuals in our cohort (4 and 11) had distinct features compared with other individuals (severe micrognathia, small mouth, downslanted palpebral fissures, and hearing loss), which might be caused by a distinct or additional mode of

pathogenicity. Larger follow-up cohort studies are needed to perform detailed genotype-phenotype correlations for *WDR5* variants to carefully characterize the complete spectrum of *WDR5*-associated phenotypes and molecular underpinnings.

In conclusion, by identifying and characterizing individuals with rare *de novo* missense variants in *WDR5*, we suggest the presence of a novel Mendelian neurodevelopmental disorder. The associated phenotype consists of ID, speech and language impairments, epilepsy, and/or ASD. In addition, a wide spectrum of associated features is reported, including but not limited to aberrant growth parameters, skeletal abnormalities, and cardiac abnormalities. More clinical and functional studies are needed for a further delineation of the full clinical and mutational spectrum and the pathogenic mechanisms associated with this disorder by combining data from clinical and experimental approaches. Based on the results of our study, we can already add *WDR5* to the growing list of human disease genes encoding COMPASS complex family subunits, such as *KMT2A* (MIM: 159555), *KMT2B* (MIM: 606834), *KMT2C* (MIM: 606833), *KMT2D* (MIM: 602133), *KDM6A* (MIM: 300128), *SETD1A* (MIM: 611052), and *SETD1B* (MIM: 611055), thereby further confirming the important role of COMPASS complex family members, and more specifically WDR5, as highly important contributors to crucial (neuro)developmental processes.

Data and code availability

The clinical dataset used for this study is included in the supplemental information (Table S1). No other datasets were generated during this study.

Supplemental information

Supplemental information can be found online at https://doi.org/10.1016/j.xhgg.2022.100157.

Acknowledgments

We thank all included individuals and their families for their contribution to this research project. We thank the late Dr. Kenneth L. Scott for providing the human *WDR5* cDNA used in this study. Funding was provided by the Netherlands Organisation for Scientific Research (NWO) Gravitation Grant 24.001.006 to the Language in Interaction Consortium (L.S.B., S.E.F., and H.G.B.), the Max Planck Society (J.d.H. and S.E.F.), and the Netherlands Organisation for Health Research and Development (ZonMw grant 91718310 to T.K.). Funding to J.M.K. was provided by a catalyst grant from the Canadian Rare Disease Models and Mechanisms Network. The research of A.C., M.I., and E.R. was supported by PROGETTEO GENE (GENE = Genomic Analysis Evaluation Network) founded by PROGETTI DI INNOVAZIONE IN AMBITO SANITARIO E SOCIO SANITARIO (BANDO EX DECRETO N. 2713 DEL 28/02/2018). A.Roos and N.K. acknowledge funding from the European Regional Development Fund (ERDF). K.Õ. and K.R. were supported by the Estonian Research Council grants PUT355 and PRG471. The Broad Center for Mendelian Genomics (UM1 HG008900) is funded by the National Human Genome Research Institute with supplemental funding provided by the National Heart, Lung, and Blood Institute under the Trans-Omics for Precision Medicine (TOPMed) program and the National Eye Institute. This work was generated within ITHACA: European Reference Network on Rare Congenital Malformations and Rare Intellectual Disability. Individuals 3 and 4 were part of the DDD study cohort, individual 6 was ascertained through the Care4Rare consortium, and individual 9 was part of the UDP-Vic program. Further information on these cohort studies is included in the supplemental information.

Declaration of interests

F.M. is a full-time employee at GeneDx, Inc. A.M.I. serves in a voluntary capacity as a member of the Human Genetics and Genomics Advances (HGG-A) Editorial board.

Received: May 23, 2022
Accepted: October 27, 2022

References

1. Copley, R.R. (2016). The unicellular ancestry of groucho-mediated repression and the origins of metazoan transcription factors. Genome Biol. Evol. *8*, 1859–1867. https://doi.org/10.1093/gbe/evw118.
2. Uhlén, M., Fagerberg, L., Hallström, B.M., Lindskog, C., Oksvold, P., Mardinoglu, A., Sivertsson, Å., Kampf, C., Sjöstedt, E., Asplund, A., et al. (2015). Proteomics. Tissue-based map of the human proteome. Science *347*, 1260419. https://doi.org/10.1126/science.1260419.
3. Guarnaccia, A.D., and Tansey, W.P. (2018). Moonlighting with WDR5: a cellular multitasker. J. Clin. Med. *7*, E21. https://doi.org/10.3390/jcm7020021.
4. Wysocka, J., Swigut, T., Milne, T.A., Dou, Y., Zhang, X., Burlingame, A.L., Roeder, R.G., Brivanlou, A.H., and Allis, C.D. (2005). WDR5 associates with histone H3 methylated at K4 and is essential for H3 K4 methylation and vertebrate development. Cell *121*, 859–872. https://doi.org/10.1016/j.cell.2005.03.036.
5. Roguev, A., Schaft, D., Shevchenko, A., Pijnappel, W.W., Wilm, M., Aasland, R., and Stewart, A.F. (2001). The Saccharomyces cerevisiae Set1 complex includes an Ash2 homologue and methylates histone 3 lysine 4. EMBO J. *20*, 7137–7148. https://doi.org/10.1093/emboj/20.24.7137.
6. Miller, T., Krogan, N.J., Dover, J., Erdjument-Bromage, H., Tempst, P., Johnston, M., Greenblatt, J.F., and Shilatifard, A. (2001). COMPASS: a complex of proteins associated with a trithorax-related SET domain protein. Proc. Natl. Acad. Sci. USA *98*, 12902–12907. https://doi.org/10.1073/pnas.231473398.
7. Cai, Y., Jin, J., Swanson, S.K., Cole, M.D., Choi, S.H., Florens, L., Washburn, M.P., Conaway, J.W., and Conaway, R.C. (2010). Subunit composition and substrate specificity of a MOF-containing histone acetyltransferase distinct from the male-specific lethal (MSL) complex. J. Biol. Chem. *285*, 4268–4272. https://doi.org/10.1074/jbc.C109.087981.
8. Suganuma, T., Gutiérrez, J.L., Li, B., Florens, L., Swanson, S.K., Washburn, M.P., Abmayr, S.M., and Workman, J.L. (2008). ATAC is a double histone acetyltransferase complex that stimulates nucleosome sliding. Nat. Struct. Mol. Biol. *15*, 364–372. https://doi.org/10.1038/nsmb.1397.

9. Ee, L.S., McCannell, K.N., Tang, Y., Fernandes, N., Hardy, W.R., Green, M.R., Chu, F., and Fazzio, T.G. (2017). An embryonic stem cell-specific NuRD complex functions through interaction with WDR5. Stem Cell Rep. *8*, 1488–1496. https://doi.org/10.1016/j.stemcr.2017.04.020.
10. Yang, Y.W., Flynn, R.A., Chen, Y., Qu, K., Wan, B., Wang, K.C., Lei, M., and Chang, H.Y. (2014). Essential role of lncRNA binding for WDR5 maintenance of active chromatin and embryonic stem cell pluripotency. Elife *3*, e02046. https://doi.org/10.7554/eLife.02046.
11. Ang, Y.S., Tsai, S.Y., Lee, D.F., Monk, J., Su, J., Ratnakumar, K., Ding, J., Ge, Y., Darr, H., Chang, B., et al. (2011). Wdr5 mediates self-renewal and reprogramming via the embryonic stem cell core transcriptional network. Cell *145*, 183–197. https://doi.org/10.1016/j.cell.2011.03.003.
12. Li, X., Li, L., Pandey, R., Byun, J.S., Gardner, K., Qin, Z., and Dou, Y. (2012). The histone acetyltransferase MOF is a key regulator of the embryonic stem cell core transcriptional network. Cell Stem Cell *11*, 163–178. https://doi.org/10.1016/j.stem.2012.04.023.
13. Ma, Z., Zhu, P., Shi, H., Guo, L., Zhang, Q., Chen, Y., Chen, S., Zhang, Z., Peng, J., and Chen, J. (2019). PTC-bearing mRNA elicits a genetic compensation response via Upf3a and COMPASS components. Nature *568*, 259–263. https://doi.org/10.1038/s41586-019-1057-y.
14. Vilhais-Neto, G.C., Fournier, M., Plassat, J.L., Sardiu, M.E., Saraf, A., Garnier, J.M., Maruhashi, M., Florens, L., Washburn, M.P., and Pourquié, O. (2017). The WHHERE coactivator complex is required for retinoic acid-dependent regulation of embryonic symmetry. Nat. Commun. *8*, 728. https://doi.org/10.1038/s41467-017-00593-6.
15. Li, Q., Mao, F., Zhou, B., Huang, Y., Zou, Z., denDekker, A.D., Xu, J., Hou, S., Liu, J., Dou, Y., and Rao, R.C. (2020). p53 integrates temporal WDR5 inputs during neuroectoderm and mesoderm differentiation of mouse embryonic stem cells. Cell Rep. *30*, 465–480.e6. https://doi.org/10.1016/j.celrep.2019.12.039.
16. Eising, E., Carrion-Castillo, A., Vino, A., Strand, E.A., Jakielski, K.J., Scerri, T.S., Hildebrand, M.S., Webster, R., Ma, A., Mazoyer, B., et al. (2019). A set of regulatory genes co-expressed in embryonic human brain is implicated in disrupted speech development. Mol. Psychiatry *24*, 1065–1078. https://doi.org/10.1038/s41380-018-0020-x.
17. Sobreira, N., Schiettecatte, F., Valle, D., and Hamosh, A. (2015). GeneMatcher: a matching tool for connecting investigators with an interest in the same gene. Hum. Mutat. *36*, 928–930. https://doi.org/10.1002/humu.22844.
18. Karczewski, K.J., Francioli, L.C., Tiao, G., Cummings, B.B., Alföldi, J., Wang, Q., Collins, R.L., Laricchia, K.M., Ganna, A., Birnbaum, D.P., et al. (2020). The mutational constraint spectrum quantified from variation in 141, 456 humans. Nature *581*, 434–443. https://doi.org/10.1038/s41586-020-2308-7.
19. Xu, C., and Min, J. (2011). Structure and function of WD40 domain proteins. Protein Cell *2*, 202–214. https://doi.org/10.1007/s13238-011-1018-1.
20. Kummeling, J., Stremmelaar, D.E., Raun, N., Reijnders, M.R.F., Willemsen, M.H., Ruiterkamp-Versteeg, M., Schepens, M., Man, C.C.O., Gilissen, C., Cho, M.T., et al. (2021). Characterization of SETD1A haploinsufficiency in humans and Drosophila defines a novel neurodevelopmental syndrome. Mol. Psychiatry *26*, 2013–2024. https://doi.org/10.1038/s41380-020-0725-5.
21. Koemans, T.S., Kleefstra, T., Chubak, M.C., Stone, M.H., Reijnders, M.R.F., de Munnik, S., Willemsen, M.H., Fenckova, M., Stumpel, C.T.R.M., Bok, L.A., et al. (2017). Functional convergence of histone methyltransferases EHMT1 and KMT2C involved in intellectual disability and autism spectrum disorder. PLoS Genet. *13*, e1006864. https://doi.org/10.1371/journal.pgen.1006864.
22. Patel, A., Dharmarajan, V., and Cosgrove, M.S. (2008). Structure of WDR5 bound to mixed lineage leukemia protein-1 peptide. J. Biol. Chem. *283*, 32158–32161. https://doi.org/10.1074/jbc.C800164200.
23. Patel, A., Vought, V.E., Dharmarajan, V., and Cosgrove, M.S. (2008). A conserved arginine-containing motif crucial for the assembly and enzymatic activity of the mixed lineage leukemia protein-1 core complex. J. Biol. Chem. *283*, 32162–32175. https://doi.org/10.1074/jbc.M806317200.
24. Dias, J., Van Nguyen, N., Georgiev, P., Gaub, A., Brettschneider, J., Cusack, S., Kadlec, J., and Akhtar, A. (2014). Structural analysis of the KANSL1/WDR5/KANSL2 complex reveals that WDR5 is required for efficient assembly and chromatin targeting of the NSL complex. Genes Dev. *28*, 929–942. https://doi.org/10.1101/gad.240200.114.
25. Odho, Z., Southall, S.M., and Wilson, J.R. (2010). Characterization of a novel WDR5-binding site that recruits RbBP5 through a conserved motif to enhance methylation of histone H3 lysine 4 by mixed lineage leukemia protein-1. J. Biol. Chem. *285*, 32967–32976. https://doi.org/10.1074/jbc.M110.159921.
26. Xue, H., Yao, T., Cao, M., Zhu, G., Li, Y., Yuan, G., Chen, Y., Lei, M., and Huang, J. (2019). Structural basis of nucleosome recognition and modification by MLL methyltransferases. Nature *573*, 445–449. https://doi.org/10.1038/s41586-019-1528-1.
27. Dou, Y., Milne, T.A., Ruthenburg, A.J., Lee, S., Lee, J.W., Verdine, G.L., Allis, C.D., and Roeder, R.G. (2006). Regulation of MLL1 H3K4 methyltransferase activity by its core components. Nat. Struct. Mol. Biol. *13*, 713–719. https://doi.org/10.1038/nsmb1128.
28. Steward, M.M., Lee, J.S., O'Donovan, A., Wyatt, M., Bernstein, B.E., and Shilatifard, A. (2006). Molecular regulation of H3K4 trimethylation by ASH2L, a shared subunit of MLL complexes. Nat. Struct. Mol. Biol. *13*, 852–854. https://doi.org/10.1038/nsmb1131.
29. Zaidi, S., Choi, M., Wakimoto, H., Ma, L., Jiang, J., Overton, J.D., Romano-Adesman, A., Bjornson, R.D., Breitbart, R.E., Brown, K.K., et al. (2013). De novo mutations in histone-modifying genes in congenital heart disease. Nature *498*, 220–223. https://doi.org/10.1038/nature12141.
30. Schuetz, A., Allali-Hassani, A., Martín, F., Loppnau, P., Vedadi, M., Bochkarev, A., Plotnikov, A.N., Arrowsmith, C.H., and Min, J. (2006). Structural basis for molecular recognition and presentation of histone H3 by WDR5. EMBO J. *25*, 4245–4252. https://doi.org/10.1038/sj.emboj.7601316.
31. Kulkarni, S.S., and Khokha, M.K. (2018). WDR5 regulates left-right patterning via chromatin-dependent and -independent functions. Development *145*, dev159889. https://doi.org/10.1242/dev.159889.
32. Firth, H.V., Richards, S.M., Bevan, A.P., Clayton, S., Corpas, M., Rajan, D., Van Vooren, S., Moreau, Y., Pettett, R.M., and Carter, N.P. (2009). DECIPHER: database of chromosomal imbalance and phenotype in humans using ensembl resources. Am. J. Hum. Genet. *84*, 524–533. https://doi.org/10.1016/j.ajhg.2009.03.010.

HGGA, Volume *4*

Supplemental information

A clustering of heterozygous missense variants in the crucial chromatin modifier WDR5 defines a new neurodevelopmental disorder

Lot Snijders Blok, Jolijn Verseput, Dmitrijs Rots, Hanka Venselaar, A. Micheil Innes, Connie Stumpel, Katrin Õunap, Karit Reinson, Eleanor G. Seaby, Shane McKee, Barbara Burton, Katherine Kim, Johanna M. van Hagen, Quinten Waisfisz, Pascal Joset, Katharina Steindl, Anita Rauch, Dong Li, Elaine H. Zackai, Sarah E. Sheppard, Beth Keena, Hakon Hakonarson, Andreas Roos, Nicolai Kohlschmidt, Anna Cereda, Maria Iascone, Erika Rebessi, Kristin D. Kernohan, Philippe M. Campeau, Francisca Millan, Jesse A. Taylor, Hanns Lochmüller, Martin R. Higgs, Amalia Goula, Birgitta Bernhard, Danita J. Velasco, Andrew A. Schmanski, Zornitza Stark, Lyndon Gallacher, Lynn Pais, Paul C. Marcogliese, Shinya Yamamoto, Nicholas Raun, Taryn E. Jakub, Jamie M. Kramer, Joery den Hoed, Simon E. Fisher, Han G. Brunner, and Tjitske Kleefstra

Supplemental information

Supplemental Material & Methods

Study participants and consent

Individuals with *WDR5* variants were identified via matchmaking using GeneMatcher[1], the Dutch genetic diagnostic variant classification database (VKGL database)[2-4], ClinVar[5] and denovo-db[6]. Clinical data and details on variants were collected in a Castor EDC database[7]. Informed consent was obtained from all participating families. For all pictures of affected individuals, specific consent to publish clinical photographs was obtained. All procedures in this study matched local ethical guidelines of the participating centres, and are in accordance with the Declaration of Helsinki.

Next generation sequencing and *in silico* variant analyses

Details on next generation sequencing methods used to identify the *WDR5* variants found in all individuals are included in table S1. Variants were analysed using Alamut Visual 2.10. Conservation was studied using a Clustal[8] alignment of WDR5 amino acid sequences extracted from Uniprot (human, mouse and C. elegans)[9]. To assess the likelihood of pathogenicity, the prediction programs SIFT[10], PolyPhen-2[11] and CADD v1.4[12] were used.

Drosophila strains and RNAi knock down assays

Flies were reared on standard cornmeal-agar media at 25°C with a 12h/12h light/dark cycle at 70% humidity. The ubiquitous driver line, *Act5C-Gal4* (stock # 4414), mushroom body driver line, *R14H06-Gal4* (stock # 48667), *UAS-mCherry-RNAi* ($mCherry^{RNAi}$ stock # 35785), and *UAS-wds-RNAi* (wds^{RNAi} stock # 32952) were obtained from the Bloomington *Drosophila* stock center. Reference and variant *UAS-WDR5::HA* transgenic flies were generated as previously described[13]. Briefly, the Gateway (Thermo Fisher Scientific) compatible WDR5 cDNA open entry clone (NCBI Acc. #: BC001635.1) in pDONR223 was shuttled to the pGW-attB-HA[14] destination vector using LR Clonase II (Thermo Fisher Scientific, Cat# 11791020) as per manufacturer's protocol. To generate WDR5 variants, site-directed mutagenesis using the Q5 site-directed mutagenesis kit (NEB - E0554S) was employed. Constructs were verified using Sanger sequencing. All *UAS-WDR5::HA*

constructs were microinjected into embryos expressing φC31 integrase with a 2nd chromosome attP docking site VK37 (PBac{y[+]-attP}VK00037)[15] and identified by *w+* (encoded by the mini-white gene in pGW-attB-HA vector).

Wds RNAi lines were validated as previously described[16; 17]. Expression of *wds^{RNAi}* with a ubiquitous *Act-Gal4* driver resulted in complete lethality and qPCR on knockdown larvae showed that the expression level of *wds* is reduced 87% when compared to controls (p=0.0023, t-test).

**Primers for generation of UAS-WDR5 variant Drosophila**<br>**Forward and Reverse Primers (5' to 3')**	
UAS-WDR5::HA$^{p.A169P}$-F	GACTTTGCCACCTCACTCGGATC
UAS-WDR5::HA$^{p.A169P}$-R	TTGAGGCACTTCCCTGTT
WDR5::HA $^{p.R196C}$-F	TGGTCTCTGTTGCATCTGGGA
WDR5::HA $^{p.R196C}$-R	TCATAGCTACTTGAAACTATCAAG
WDR5::HA $^{p.A201V}$-F	TGGGACACCGTCTCGGGCCAG
WDR5::HA $^{p.A201V}$-R	GATGCGACAGAGACCATCATAGCTACTTGAAAC
WDR5::HA $^{p.T208M}$-F	TGCCTGAAGATGCTCATCGATGACGAC
WDR5::HA $^{p.T208M}$-R	CTGGCCCGAGGCGGTGTC
WDR5::HA $^{p.D213N}$-F	CATCGATGACAACAACCCCCC
WDR5::HA $^{p.D213N}$-R	AGCGTCTTCAGGCACTGG
WDR5::HA $^{p.K245R}$-F	GACTACAGCAGGGGGAAGTGC
WDR5::HA $^{p.K245R}$-R	CCAGAGCTTCAGAGTGTTG

Drosophila memory assays

Short-term memory (STM) and long-term memory (LTM) was assessed using courtship conditioning, as previously described[18-20]. Briefly, for each fly pair a courtship index (CI) was calculated, which is the proportion of time spent courting over 10 min. The memory index (MI) represents the percentage reduction in courtship behaviour in trained flies compared to naive and is used to compare memory between different genotypes. MI was calculated using the formula: MI = ($\bar{x}$ $CI_{naïve}$ - $\bar{x}$ $CI_{trained}$) / $\bar{x}$ $CI_{naïve}$. Statistics were generated as previously described[18; 20].

Western Blotting

For the western blot with lysates from *UAS-WDR5::HA* Drosophila, protein was extracted from 10 adult flies expressing *UAS-WDR5::HA* reference and variant transgenes via the ubiquitous *Act-Gal4* driver. Western blotting was preformed according to standard protocols using rabbit anti-HA (1:1000; Cell Signaling Technology C29F4), mouse anti-β-tubulin (1:5000; Developmental Studies Hybridoma Bank [DSHB]) primary antibodies, and horseradish-peroxidase-conjugated secondary antibodies goat anti-rabbit (1:3000; Bio-Rad 170-6515) and goat anti-mouse (1:3000; Bio-Rad 170-6516).

For the western blot with lysates from HEK293/T17 cells, whole-cell lysates were collected by treatment with RIPA buffer (Cell Signalling) supplemented with 1% PMSF and protease inhibitor cocktail (Roche). Samples were incubated for 20 min at 4 °C followed by centrifugation for 30 min at 12,000 rpm at 4 °C. Proteins were resolved on 4–15% Mini-PROTEAN TGX Precast Gels (Bio-Rad) and transferred onto polyvinylidene fluoride membranes using a TransBlot Turbo Blotting system (Bio-Rad). Membranes were blocked in 5% milk for 1 h at room temperature and then probed with mouse-anti-EGFP (1:8000; Clontech, 632380). Next, membranes were incubated with HRP-conjugated goat-anti-mouse IgG (1:10,000; Jackson ImmunoResearch) for 1 h at room temperature. Bands were visualized with Novex ECL Chemiluminescent Substrate Reagent (Invitrogen) using a ChemiDoc XRS + System (Bio-Rad). Equal protein loading was confirmed by probing with mouse-anti-β-actin antibody (1:10,000; Sigma, A5441).

DNA expression constructs for cell based assays and site-directed mutagenesis

WDR5 (NM_017588.3) and RbBP5 (NM_005057.4) coding sequences were amplified using primers listed below. cDNAs were subcloned using *HindIII/XbaI* (WDR5) and *SalI*/*BamHI* (RbBP5) restriction sites into pRluc and pYFP, created by modification of the pEGFP-C2 vector (Clontech) as described before.[21] Variants in WDR5 were generated using the QuikChange Lightning Site-Directed Mutagenesis Kit (Agilent). The primers used for site-directed mutagenesis are listed below.

**Primers for amplifying and subcloning of WDR5 and RbBP5 cDNAs**	
WDR5-cloning-F	AGAATTAAGCTTGTTGGCGACGGAGGAGAAGAA
WDR5-cloning-R	GGATCCTCTAGATTAGCAGTCACTCTTCCACA
RBBP5-cloning-F1	GACGATGTCGACTGCTGAACCTCGAGTTGCTGGA
RBBP5-cloning -R1	TCCGGTGGATCCTCATAACAGTTCTGAGATTG

**Primers used for site-directed mutagenesis**	
WDR5-R196C-R	CAAGTAGCTATGATGGTCTCTGTTGCATCTGGGACA
WDR5-R196C-F	TGTCCCAGATGCAACAGAGACCATCATAGCTACTTG
WDR5-A201V-R	ATCTGGGACACCGTCTCAGGCCAGTGC
WDR5-A201V-F	GCACTGGCCTGAGACGGTGTCCCAGAT
WDR5-T208M-R	AGGCCAGTGCCTGAAGATGCTCATCGATGAC
WDR5-T208M-F	GTCATCGATGAGCATCTTCAGGCACTGGCCT

Cell culture

HEK293T/17 cells (CRL-11268, ATCC) were cultured in DMEM supplemented with 10% fetal bovine serum and 1x penicillin-streptomycin (all Invitrogen) at 37°C with 5% CO_2. Transfections were performed using GeneJuice (Millipore) following the manufacturer's protocol.

Fluorescence microscopy

HEK293T/17 cells were grown on poly-D-lysine (Sigma) coated coverslips. Cells were fixed with 4% paraformaldehyde (PFA, Electron Microscopy Sciences) 48 h after transfection with YFP-tagged WDR5 variants. Nuclei were stained with Hoechst 33342 (Invitrogen). Fluorescence images were acquired with a Zeiss LSM880 confocal microscope and Airyscan detector, with a 2.0 zoom factor using ZEN Image Software (Zeiss).

Three-dimensional (3D) protein modelling

The effects of the identified variants on the WDR5 protein and its interaction with other proteins in the COMPASS family complexes were analyzed using YASARA View[22] with FoldX v4.0 plugin[23]. For the WDR5 structure, PDB file 2GNQ was used. PDB files 6KIV and 6KIW[24] were used for the analysis of the core COMPASS complexes, respectively; the 6UH5 file[25] of the yeast COMPASS model was used for the comparison with the human COMPASS complex. To optimize the position of amino-acid sidechains, all the PDB files that were used were corrected by the FoldX repair function using default settings. Different protein structures were aligned with SHEBA procedure[26], as implemented in YASARA.

BRET assay

BRET assays were performed as previously described [21]. HEK293T/17 cells were transfected in white clear-bottomed 96-well plates with increasing molar ratios of YFP-fusion proteins and constant amounts of Rluc-fusion proteins (donor/acceptor ratios of 1/0.5, 1/1, 1/2, 1/3, 1/6, 1/9). YFP and Rluc fused to a C-terminal nuclear localization signal were used as control proteins. After 48 h, medium was replaced with phenol red-free DMEM, supplemented with 10% fetal bovine serum (both Invitrogen), containing 60 µM EnduRen Live Cell Substrate (Promega). After incubation for 4 h at 37 °C, measurements were taken in live cells with an Infinite M200PRO Microplate reader (Tecan) using the Blue1 and Green1 filters. Corrected BRET ratios were calculated with the following formula: [Green1($_{\text{experimental condition}}$)/Blue1($_{\text{experimental condition}}$)] – [Green1($_{\text{control condition}}$)/Blue1($_{\text{control condition}}$)], with only the Rluc control protein expressed in the

control condition. Curve fitting was done with a non-linear regression equation assuming a single binding site (y = BRETmax * x / (BRET50 / x) using GraphPad Prism Software.

Figure S1: Immunoblot analysis with lysates from HEK293/T17 cells

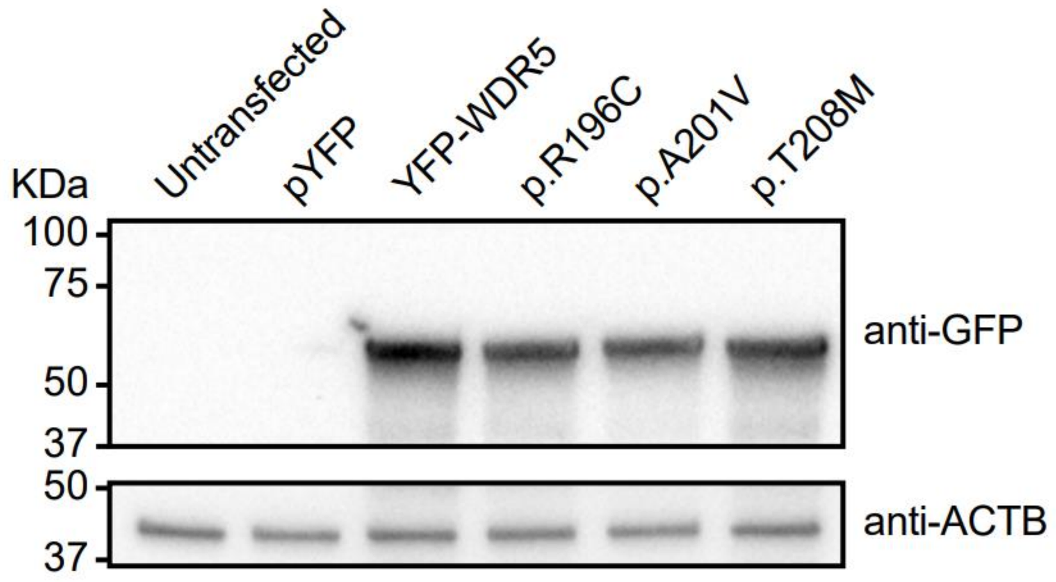

Immunoblot of whole-cell lysates (HEK293/T17 cells) expressing YFP-tagged WDR5 variants probed with anti-EGFP antibody. Expected molecular weight for all variants is ~65 kDa. The blot was stripped and probed for β-actin to ensure equal protein loading.

Figure S2: MetaDome intolerance visualization of WDR5

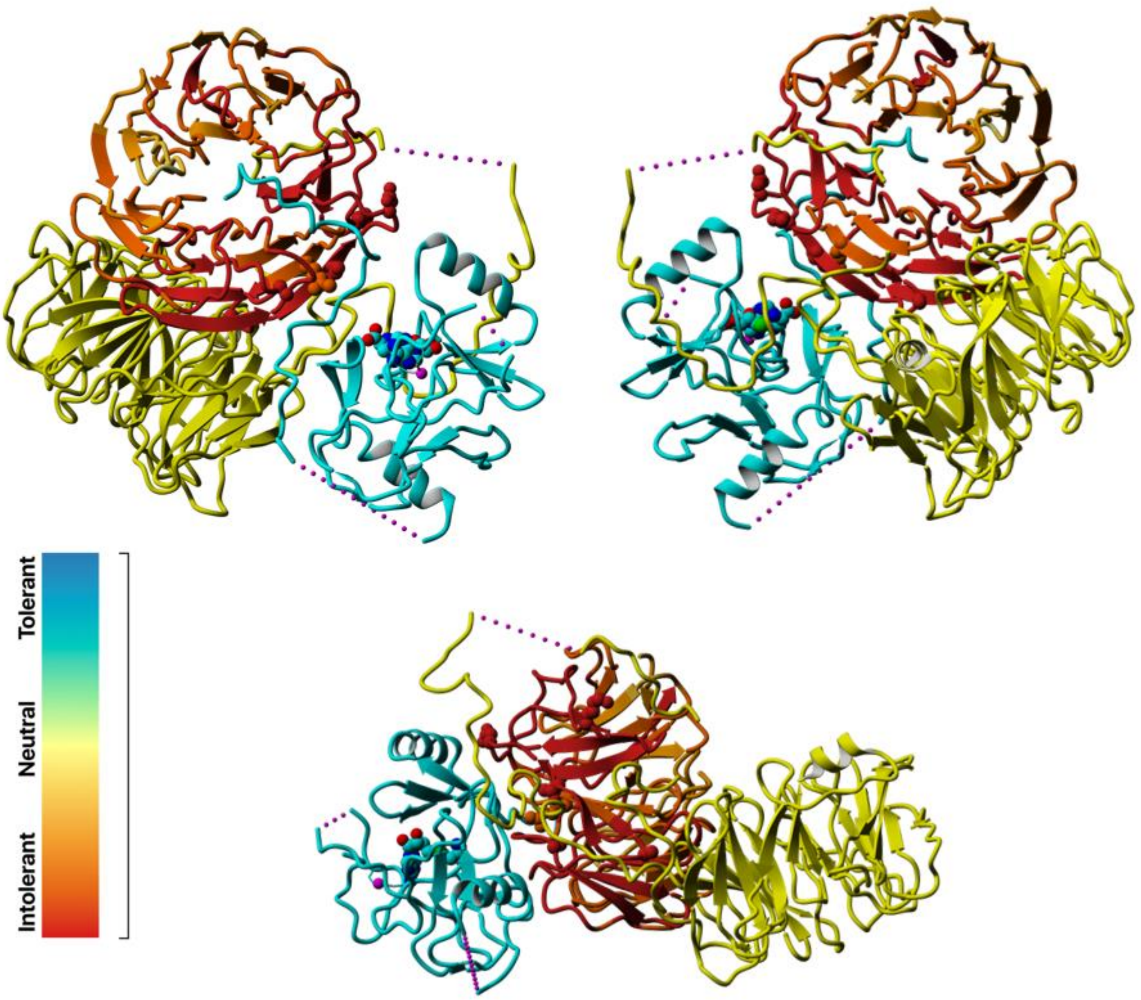

WDR5 is coloured in line with the MetaDome tolerance scale shown. RbBP5 is shown in yellow and KMT2A in cyan (PDB:6KIV). As can be seen in this figure, WDR5 is generally intolerant to missense variants, but WDR5 amino acids that are known to interact with other proteins are most intolerant (darker red).

Figure S3: Comparison of the core human KMT2A with the yeast COMPASS complexes

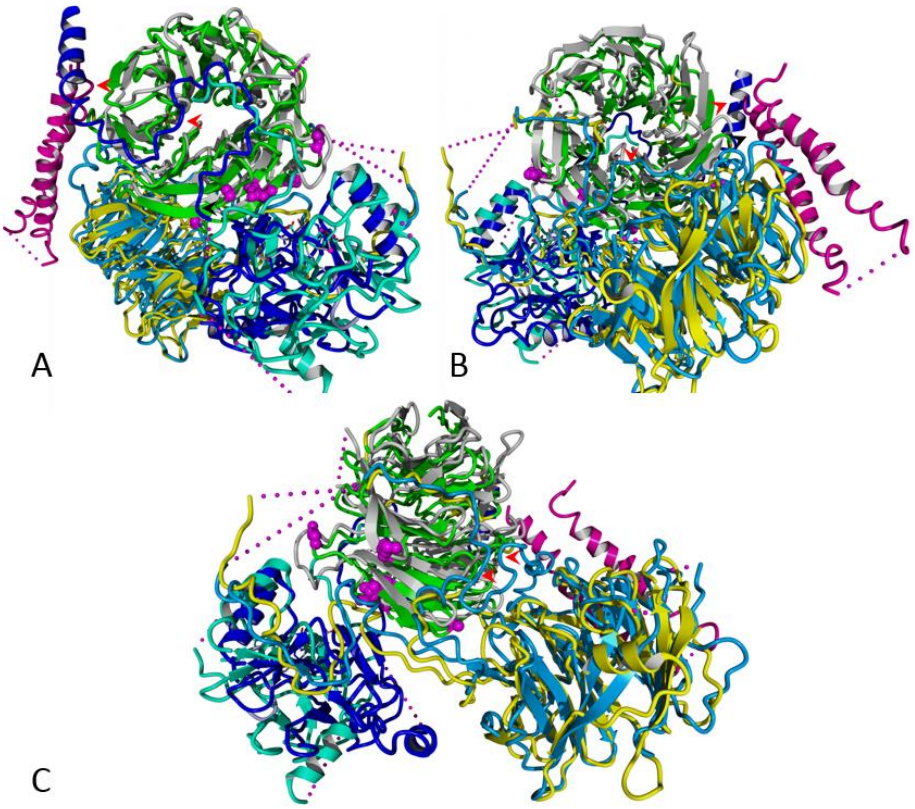

The alignment of human WDR5 in complex with RbBP5 and KMT2A from the core COMPASS complex (PDB:6KIV) with homologues of the yeast COMPASS complex (PDB:6UH5) is shown: WDR5 (green, p.33-332) with its homologue Swd3 (grey, p.16-326); RbBP5 (yellow, p.1-380) with its homologue Swd1 (light blue, p.1-435); KMT2A (cyan, p.3764-3969) with yeast homologue Set1c (dark blue, p.819-999). Additionally, yeast Spp1 (purple) is shown. The Spp1 homologue is not present in human COMPASS family complexes. The locations of the amino acids that are affected in patients identified in this study are shown with balls (magenta). Three different angles are shown: WDR5 faced from the WIN site (A), from the WBM site (B), and from the side between WIN and WBM (C).

The human core COMPASS/COMPASS family complexes (eg., KMT2A) are highly conserved and have a structure similar to the yeast COMPASS complex. Because the yeast COMPASS complex proteins in the 3D model are more complete, substantially more extensive interaction of the RbBP5 and KMT2A homologues with WDR5 homologues can be observed (red arrows). Additionally, another interaction site of the WDR5 homologue is observed with a Spp1 protein

These 3D modelling data, in addition to the high conservation level and low tolerance to the missense and LoF variants in the general population, suggest that also human WDR5 may have significantly more extensive interaction surfaces within COMPASS family complexes and other chromatin-remodelling complexes.

Supplemental note 1: Further details on individual 12 with c.742-2del (p.?) variant

Facial features of individual 12 with a *de novo* c.742-2del (p.?) variant

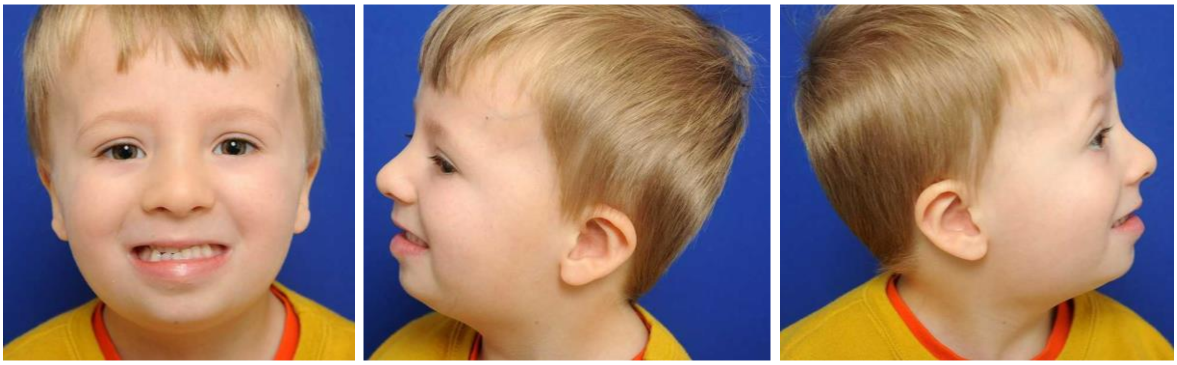

Facial photographs of individual 12 at age 4y1m

Variant analysis using splice prediction programs for c.742-2del (p.?) variant

Prediction program	Possible effect of c.742-2del variant
SpliceSiteFinder-like	Loss of acceptor site (exon 12), possible creation of alternative acceptor site 9bp upstream, resulting in in-frame deletion of 3AA (p.(Cys248_Lys250del)?
MaxEntScan	Loss of acceptor site (exon 12), possible creation of alternative acceptor site 9bp upstream, resulting in in-frame deletion of 3AA (p.(Cys248_Lys250del)?
GeneSplicer	Loss of acceptor site (exon 12), possible creation of alternative acceptor site 9bp upstream, resulting in in-frame deletion of 3AA (p.(Cys248_Lys250del)?
NNSPLICE	Loss of acceptor site (exon 12), skipping of exon 12?
SpliceAI	Loss of acceptor site (exon 12) resulting in skipping of exon 12 or creation of an alternative acceptor site 100bp downstream?

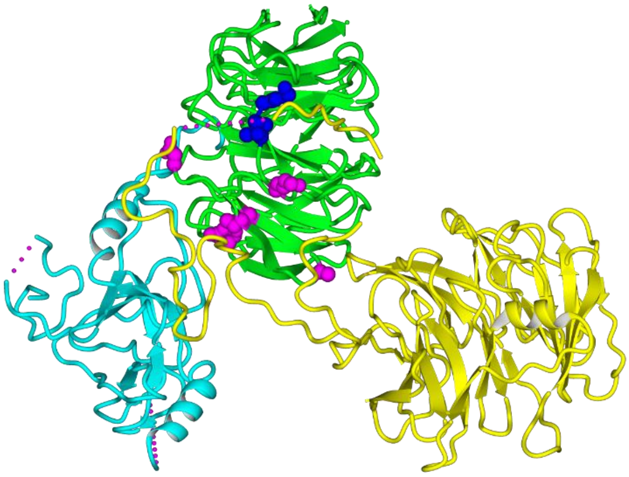

Three-dimensional analysis of c.742-2del variant and missense variants in WDR5

The WDR5 protein (green) in interaction with RbBP5 (yellow) and KMT2A/MLL1 (cyan) (PDB:6KIV). The location of amino acid substitutions (as the result of missense variants in our study) is shown in red. Amino acids involved in the possible in-frame deletion p.(Cys248_Lys250del) are depicted in dark blue.

Supplemental note 2: Detailed description and visualization of the predicted effect of identified WDR5 variants

p.(Ala169Pro)

Wild type residue role	Effect of the residue substitution
Ala169 is located in a turn from the third to fourth WDR5 beta-propeller. Despite the fact that the Ala169 is located in close proximity to the KMT2A, and KMT2C interaction sites, it does not directly interact with the KMT enzymes.	Change from the alanine to a larger proline at this position is predicted to result in a local backbone change, because of the rigid sidechain of the proline. This change is predicted to disturb the flexibility and local structure of WDR5, which will disrupt the binding to the KMT enzymes.

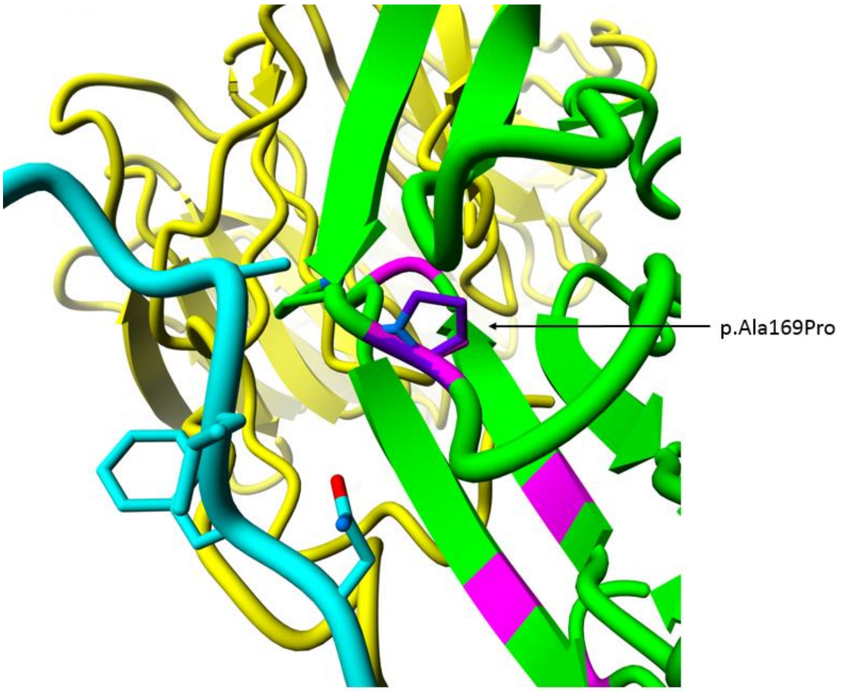

WDR5 (green) interaction with KMT2A (cyan) and RbBP5 (yellow), are shown from the core COMPASS complex (PDB:6KIV). The mutated aminoacid and nearby aminoacids are shown with sticks. The wild type alanine at the position p.169 is colored in magenta and the mutated proline in purple.

p.(Arg196Cys)

Wild type residue role	Effect of the residue substitution
Arg196 is located on the WDR5 lateral surface for interaction with RbBP5 and KMT2A enzymes. Arg196 interacts with Asn3779 in the KMT2A protein and Phe332 in the C-term tail of RbBBP5 but has no visible interactions with the KMT2C protein.	Cysteine is a much smaller residue and does not have a charge. Therefore, a change from the arginine to cysteine at this position would result in a loss of the hydrogen-bond with Asn3779 in KMT2A, as well resulting in an empty pocket between the WDR5, KMT2A and RbBBP5 interaction surfaces, which would lead to a loss of packing interactions and disruption of the interactions between the proteins.

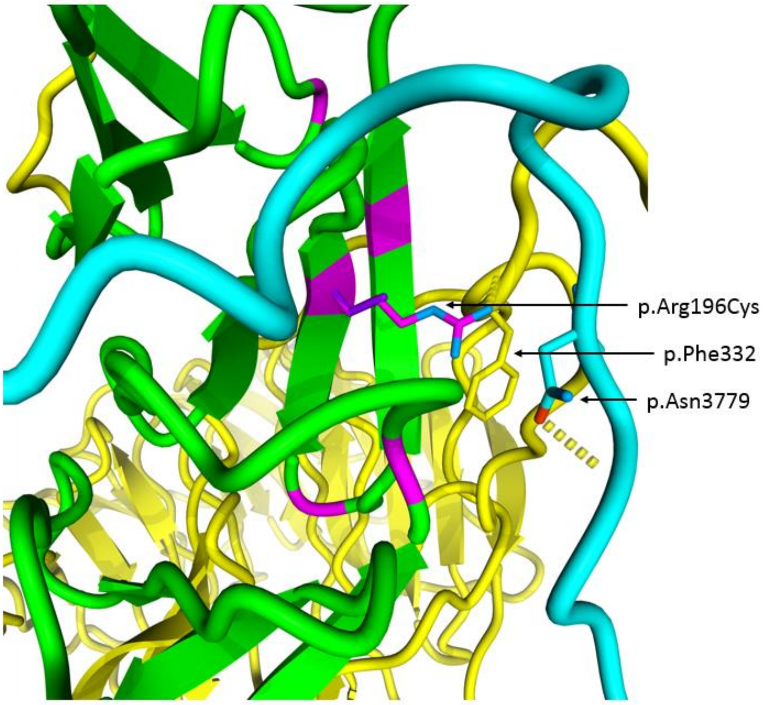

WDR5 (green) interaction with KMT2A (cyan) and RbBP5 (yellow), are shown from the core COMPASS complex (PDB:6KIV). The mutated aminoacid and nearby aminoacids are shown with sticks. The wild type arginine at the position p.196 is colored in magenta and the mutated cysteine in purple.

p.(Ala201Val)

Wild type residue role	Effect of the residue substitution
Ala201 is located on the WBM surface of WDR5 and interacts with RbBP5. It is located in clear proximity to Arg56 and Leu54 in the RbBP5 protein.	Despite the fact that valine is also small and non-polar, it has a bigger sidechain than alanine. Therefore, change to a valine at this position could affect the interaction with RbBP5 because of the change of the interaction surface.

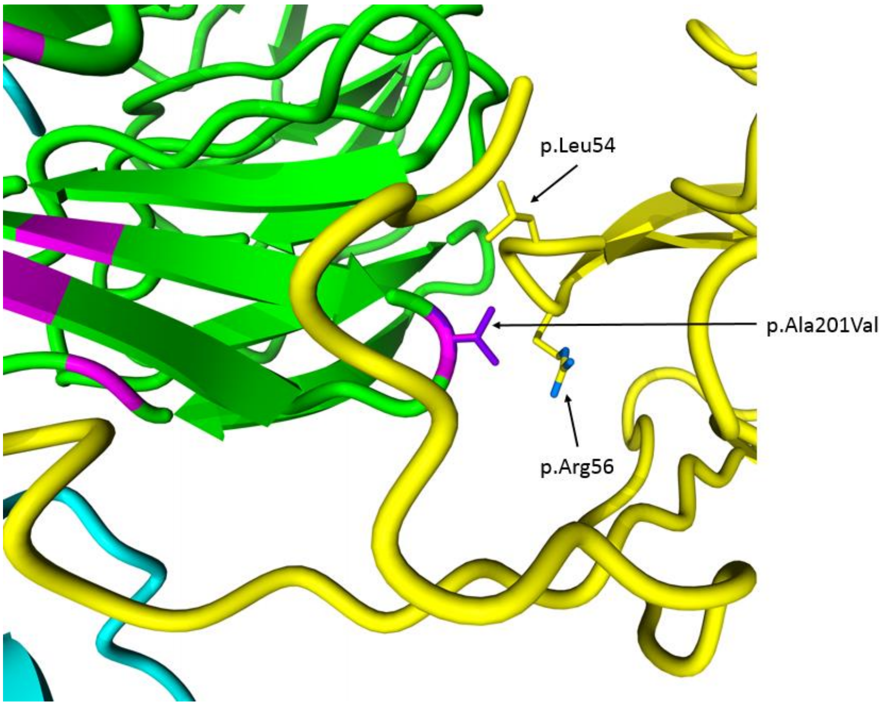

WDR5 (green) interaction with KMT2A (cyan) and RbBP5 (yellow), are shown from the core COMPASS complex (PDB:6KIV). The mutated aminoacid and nearby aminoacids are shown with sticks. The wild type alanine at the position p.201 is colored in magenta and the mutated valine in purple.

p.(Thr208Met)

Wild type residue role	Effect of the residue substitution
Thr208 in WDR5 interacts with several RbBP5 C-term tail amino acids (Ala331, Pro334). Additionally, it makes a hydrogen-bond with a backbone of Ala331 in RbBBP5.	Methionine has a substantially bigger size than threonine and is not able to form the hydrogen-bond with RbBP5 Ala331. Therefore, a change to methionine at this position is expected to disrupt the WDR5 interaction interface with RbBP5.

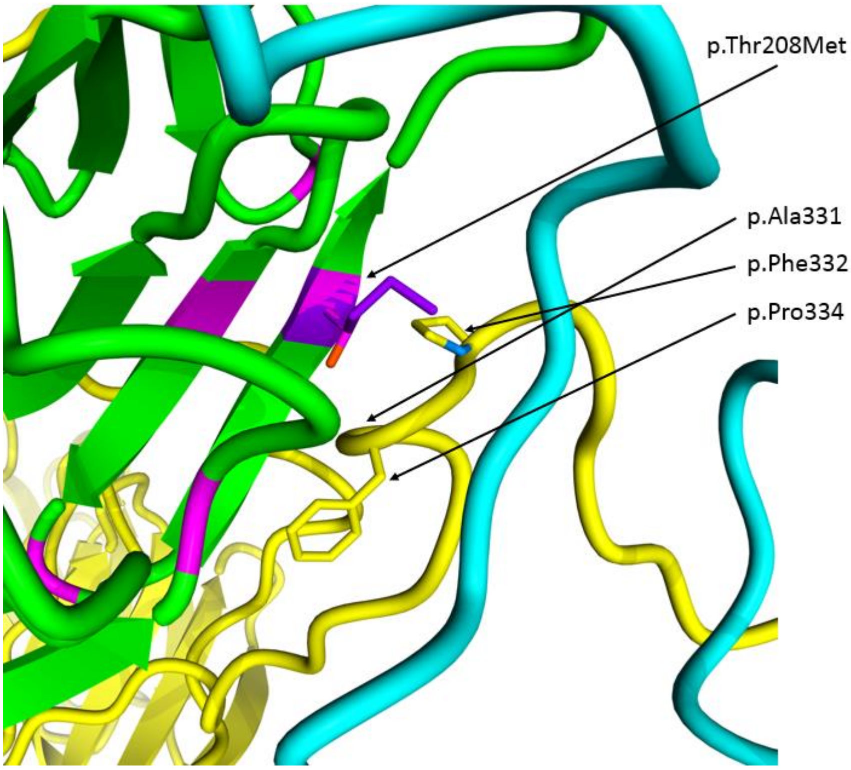

WDR5 (green) interaction with KMT2A (cyan) and RbBP5 (yellow), are shown from the core COMPASS complex (PDB:6KIV). The mutated aminoacid and nearby aminoacids are shown with sticks. The wild type threonine at the position p.208 is colored in magenta and the mutated methionine in purple.

p.(Asp213Asn)

Wild type residue role	Effect of the residue substitution
Asp213 is located in a WDR5 hydrophylic loop, which is involved in the interaction with the KMT enzymes. Although located distantly, it may interact with a positively charged KMT2A Lys3878, because the lysine has a highly flexible sidechain.<br><br>Additionally, Asp213 forms a hydrogen-bond with Asn235 in WDR5.	Change to the aspartate would result in a similar amino acid with similar size, although the negative charge of the aspartic acid would be lost. The hydrogen bond with WDR5 Asn235 would be lost due to this change, which may disrupt the stability and position of the loop, and, therefore, affect interaction with the KMT enzymes. Additionally, it can lose interactions with positively charged KMT residues. However, the exact effect of the variant is unknown.

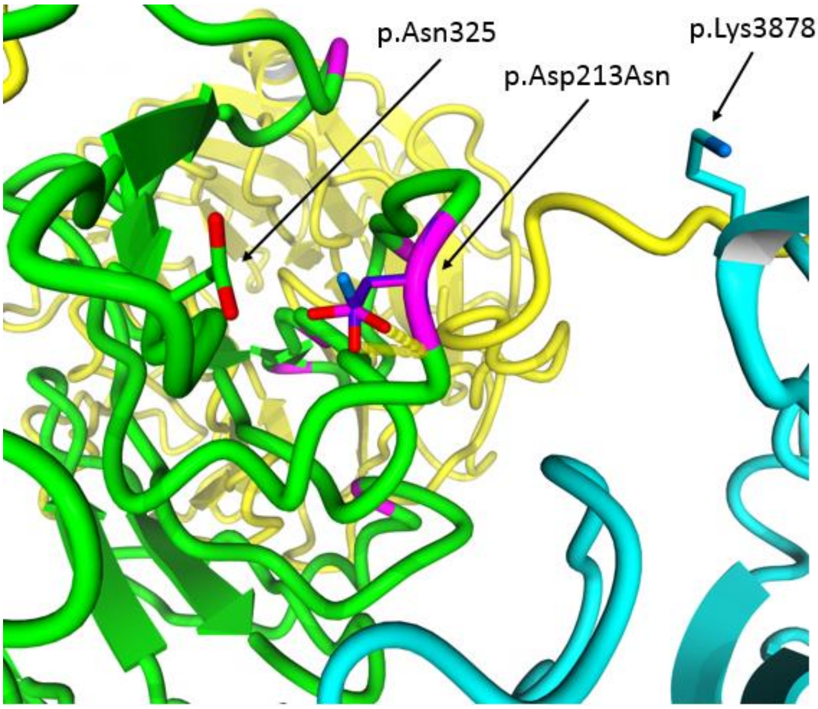

WDR5 (green) interaction with KMT2A (cyan) and RbBP5 (yellow) are shown from the core COMPASS complexes (PDB:6KIV and 6KIW, respectively). The mutated aminoacid and nearby aminoacids are shown with sticks. The wild type aspartic acid at the position p.213 is colored in magenta and the mutated aspartate in purple.

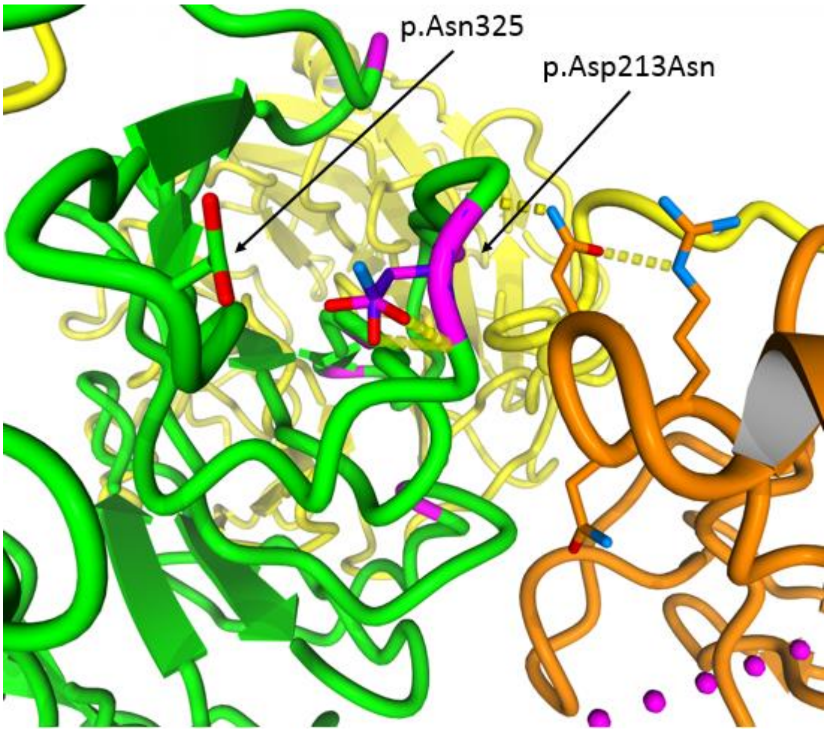

WDR5 (green) interaction with KMT2C (orange) and RbBP5 (yellow) are shown from the core COMPASS complexes (PDB:6KIV and 6KIW, respectively). The mutated aminoacid and nearby aminoacids are shown with sticks. The wild type aspartic acid at the position p.213 is colored in magenta and the mutated aspartate in purple.

p.(Lys245Arg)

Wild type residue role	Effect of the residue substitution
Lys245 located in a position that is a significant distance from the site of interaction with RbBP5 and KMT enzymes and is not known to be involved in a protein interaction.	A change from lysine to arginine at this position, would result in a similar amino acid by charge and flexibility of the side-chain with minimal effect on protein structure or interactions. Even though arginine is slightly larger, the effect of this variant is not clear.

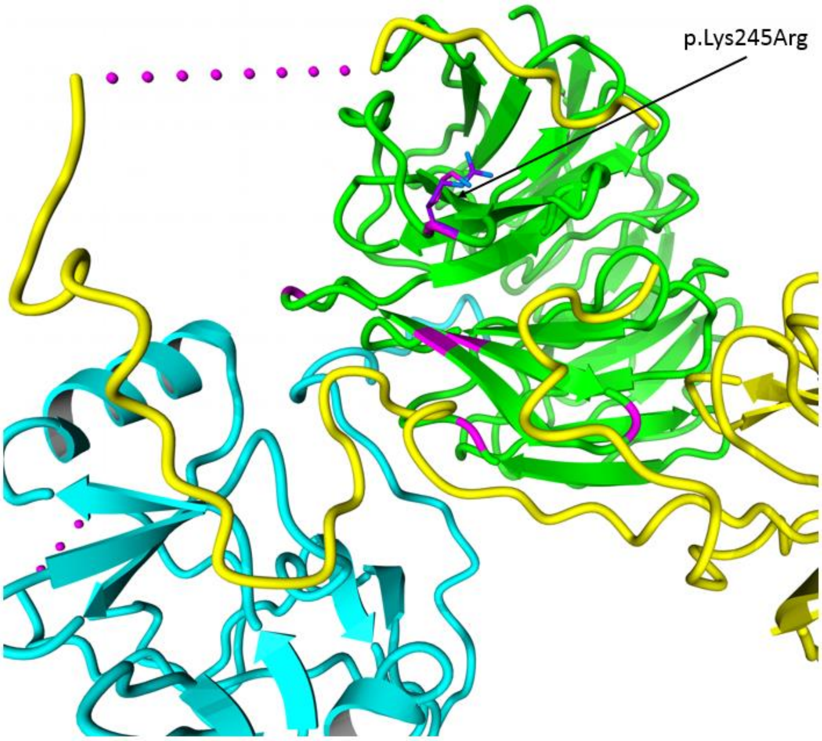

WDR5 (green) interaction with KMT2A (cyan) and RbBP5 (yellow), are shown from the core COMPASS complex (PDB:6KIV). The mutated aminoacid and nearby aminoacids are shown with sticks. The wild type lysine at the position p.245 is colored in magenta and the mutated arginine in purple.

Supplemental acknowledgements

Individuals 3 and 4 in this study were part of the DDD study cohort. The DDD study presents independent research commissioned by the Health Innovation Challenge Fund [grant number HICF-1009-003]. This study makes use of DECIPHER (http://decipher.sanger.ac.uk), which is funded by Wellcome. See Nature PMID: 25533962 or www.ddduk.org/access.html for full acknowledgement. Individual 6 was ascertained through the Care4Rare consortium. Individual 9 was part of the Undiagnosed Diseases Program Victoria (UDP-Vic). UDP-Vic acknowledges financial support from the Murdoch Children’s Research Institute and the Harbig Foundation. The research conducted at the Murdoch Children’s Research Institute was supported by the Victorian Government's Operational Infrastructure Support Program. Sequencing and analysis were provided by the Broad Institute of MIT and Harvard Center for Mendelian Genomics (Broad CMG) and was funded by the National Human Genome Research Institute, the National Eye Institute, and the National Heart, Lung and Blood Institute grant UM1 HG008900 and in part by National Human Genome Research Institute grant R01 HG009141.

References

1. Sobreira, N., Schiettecatte, F., Valle, D., and Hamosh, A. (2015). GeneMatcher: a matching tool for connecting investigators with an interest in the same gene. Hum Mutat 36, 928-930.
2. van der Velde, K.J., Imhann, F., Charbon, B., Pang, C., van Enckevort, D., Slofstra, M., Barbieri, R., Alberts, R., Hendriksen, D., Kelpin, F., et al. (2019). MOLGENIS research: advanced bioinformatics data software for non-bioinformaticians. Bioinformatics 35, 1076-1078.
3. Swertz, M.A., Dijkstra, M., Adamusiak, T., van der Velde, J.K., Kanterakis, A., Roos, E.T., Lops, J., Thorisson, G.A., Arends, D., Byelas, G., et al. (2010). The MOLGENIS toolkit: rapid prototyping of biosoftware at the push of a button. BMC Bioinformatics 11 Suppl 12, S12.
4. Swertz, M.A., and Jansen, R.C. (2007). Beyond standardization: dynamic software infrastructures for systems biology. Nat Rev Genet 8, 235-243.
5. Landrum, M.J., Lee, J.M., Benson, M., Brown, G.R., Chao, C., Chitipiralla, S., Gu, B., Hart, J., Hoffman, D., Jang, W., et al. (2018). ClinVar: improving access to variant interpretations and supporting evidence. Nucleic Acids Res 46, D1062-D1067.
6. Scholte, E., Van Duijn, E., Dijkxhoorn, Y., Noens, I., and Van Berckelaer-Onnes, I. (2008). Vineland screener 0–6 years: manual of the Dutch adaptation. PITS, Leiden.
7. Castor, E.D.C. (2019). Castor Electronic Data Capture. In. (
8. Sievers, F., Wilm, A., Dineen, D., Gibson, T.J., Karplus, K., Li, W., Lopez, R., McWilliam, H., Remmert, M., Söding, J., et al. (2011). Fast, scalable generation of high-quality protein multiple sequence alignments using Clustal Omega. In Mol Syst Biol. p 539.
9. The Uniprot Consortium (2018). UniProt: a worldwide hub of protein knowledge. Nucleic Acids Res 47, D506-D515.
10. Vaser, R., Adusumalli, S., Leng, S.N., Sikic, M., and Ng, P.C. (2016). SIFT missense predictions for genomes. Nat Protoc 11, 1-9.
11. Adzhubei, I.A., Schmidt, S., Peshkin, L., Ramensky, V.E., Gerasimova, A., Bork, P., Kondrashov, A.S., and Sunyaev, S.R. (2010). A method and server for predicting damaging missense mutations. Nat Methods 7, 248-249.
12. Kircher, M., Witten, D.M., Jain, P., O'Roak, B.J., Cooper, G.M., and Shendure, J. (2014). A general framework for estimating the relative pathogenicity of human genetic variants. Nature genetics 46, 310-315.
13. Harnish, J.M., Deal, S.L., Chao, H.T., Wangler, M.F., and Yamamoto, S. (2019). In Vivo Functional Study of Disease-associated Rare Human Variants Using Drosophila. J Vis Exp.
14. Bischof, J., Bjorklund, M., Furger, E., Schertel, C., Taipale, J., and Basler, K. (2013). A versatile platform for creating a comprehensive UAS-ORFeome library in Drosophila. Development 140, 2434-2442.
15. Venken, K.J., He, Y., Hoskins, R.A., and Bellen, H.J. (2006). P[acman]: a BAC transgenic platform for targeted insertion of large DNA fragments in D. melanogaster. Science 314, 1747-1751.
16. Mainland, R.L., Lyons, T.A., Ruth, M.M., and Kramer, J.M. (2017). Optimal RNA isolation method and primer design to detect gene knockdown by qPCR when validating Drosophila transgenic RNAi lines. BMC Res Notes 10, 647.
17. Chubak, M.C., Nixon, K.C.J., Stone, M.H., Raun, N., Rice, S.L., Sarikahya, M., Jones, S.G., Lyons, T.A., Jakub, T.E., Mainland, R.L.M., et al. (2019). Individual components of the SWI/SNF chromatin remodelling complex have distinct roles in memory neurons of the Drosophila mushroom body. Dis Model Mech 12.
18. Koemans, T.S., Oppitz, C., Donders, R.A.T., van Bokhoven, H., Schenck, A., Keleman, K., and Kramer, J.M. (2017). Drosophila Courtship Conditioning As a Measure of Learning and Memory. J Vis Exp.

19. Siegel, R.W., and Hall, J.C. (1979). Conditioned responses in courtship behavior of normal and mutant Drosophila. Proc Natl Acad Sci U S A 76, 3430-3434.
20. Kummeling, J., Stremmelaar, D.E., Raun, N., Reijnders, M.R.F., Willemsen, M.H., Ruiterkamp-Versteeg, M., Schepens, M., Man, C.C.O., Gilissen, C., Cho, M.T., et al. (2021). Characterization of SETD1A haploinsufficiency in humans and Drosophila defines a novel neurodevelopmental syndrome. Mol Psychiatry 26, 2013-2024.
21. Deriziotis, P., Graham, S.A., Estruch, S.B., and Fisher, S.E. (2014). Investigating protein-protein interactions in live cells using bioluminescence resonance energy transfer. J Vis Exp.
22. Krieger, E., and Vriend, G. (2014). YASARA View—molecular graphics for all devices—from smartphones to workstations. Bioinformatics 30, 2981-2982.
23. Schymkowitz, J., Borg, J., Stricher, F., Nys, R., Rousseau, F., and Serrano, L. (2005). The FoldX web server: an online force field. Nucleic acids research 33, W382-W388.
24. Xue, H., Yao, T., Cao, M., Zhu, G., Li, Y., Yuan, G., Chen, Y., Lei, M., and Huang, J. (2019). Structural basis of nucleosome recognition and modification by MLL methyltransferases. Nature 573, 445-449.
25. Hsu, P.L., Shi, H., Leonen, C., Kang, J., Chatterjee, C., and Zheng, N. (2019). Structural Basis of H2B Ubiquitination-Dependent H3K4 Methylation by COMPASS. Mol Cell 76, 712-723.e714.
26. Jung, J., and Lee, B. (2000). Protein structure alignment using environmental profiles. Protein Engineering, Design and Selection 13, 535-543.
